# Baicalein analogues as prospective SARS-CoV-2 main protease (M^pro^) inhibitors: A dataset of molecular docking-based virtual screening hits

**DOI:** 10.1016/j.dib.2024.110618

**Published:** 2024-06-12

**Authors:** Qiao Jie Wong, Zhe Hong Low, Zi Yue Chan, Vasudeva Rao Avupati

**Affiliations:** a Department of Biomedical Science, School of Health Sciences, IMU University (Formerly known as International Medical University), Kuala Lumpur 57000, Malaysia; b Department of Pharmaceutical Chemistry, School of Pharmacy, IMU University (Formerly known as International Medical University), Kuala Lumpur 57000, Malaysia; c Centre for Bioactive Molecules & Drug Delivery, Institute for Research, Development and Innovation, IMU University (Formerly known as International Medical University), Kuala Lumpur 57000, Malaysia

**Keywords:** Baicalein analogues dataset, SARS-CoV-2 main protease inhibitors, Virtual screening, Molecular docking

## Abstract

The global coronavirus disease 2019 (COVID-19) pandemic originating from severe acute respiratory syndrome coronavirus 2 (SARS-CoV-2) has exerted profound damage to millions of lives. Baicalein is a flavonoid that has gotten a lot of attention as a possible SARS-CoV-2 main protease (M^pro^) inhibitor because it can fight off many different viruses. We prepared and screened three sets of databases, each containing 2563 baicalein analogues, against M^pro^ using molecular docking simulation. The data showed that several baicalein analogues exhibited stable binding energies relative to standard baicalein, indicating that they have some selectivity against M^pro^. The binding properties of the top three stable analogues from each database were further analyzed with respect to their binding properties, such as binding mode, binding energy, and binding interaction of putative stable ligand confirmations at the target binding site region.

Specifications Table

**Table:** 

Subject	Pharmaceutical Sciences – Computer-Aided Drug Design (CADD)
Specific subject area	Structure-Based Drug Desing (SBDD) and virtual screening of databases of baicalein analogues as prospective SARS-CoV-2 Main Protease (M^pro^) Inhibitors.
Type of data	Raw data of tables and Images
Data collection	Molecular docking-based identification of virtual drug-like molecules by using ligand design and molecular docking-based virtual screening.
Data source location	Institution: School of Pharmacy, IMU University (Formerly known as International Medical University) City/Town/Region: Kuala Lumpur Country: Malaysia
Data accessibility	Wong, Qiao Jie; Low, Zhe Hong; Chan, Zi Yue; Avupati, Vasudeva Rao (2024), “Ligand-Target Interaction Diagrammes: Baicalein Analogues as SARS-CoV-2 Main Protease (M^pro^) Inhibitors”, Mendeley Data, V2, doi: 10.17632/xd4njjngpn.2 Link: https://data.mendeley.com/datasets/xd4njjngpn/2
Related research article	None

## 1 Value of the Data

• The data presented in this article is of greater importance for the design, discovery, and development of new drugs to fight against various multifactorial viral infections.
• The structure-activity relationship (SAR) summaries discussed for each of the studies reported in the literature are useful to medical chemists in the field of antiviral drug discovery and development.
• The acquired data could be useful to explore the importance of various chemical substitutions on rhodanine to develop a bioactive ligand against a specific virus.
• The acquired data also helps researchers to review the role of rhodanine and its derivatives in the design of new rhodanine-based antiviral drug-like compounds for the reason that the data presented is very specific and curated to rhodanine analogues as antiviral agents.
• The acquired data revealed the variability of chemical structural features with respect to their SAR against a specific virus; these variations provided deeper insight for a medicinal chemist to design novel antiviral therapeutics with more selectivity and intrinsic activity. Furthermore, this article also helps to design broad-spectrum antiviral agents that retain key pharmacophores responsible for targeting common structural proteins of viruses.

## 2 Background

Severe Acute Respiratory Syndrome Coronavirus 2 (SARS-CoV-2) is a causative agent of the global pandemic coronavirus disease 2019 (COVID-19) [1]. Main protease (M^pro^) has been identified as one of the potential drug targets to discover anti-COVID drugs, it is a cysteine protease that plays a pivotal role in the viral replication by interfering the cleavage of polyproteins essential for the assembly of viral replication-transcription [2]. Flavonoids are natural products abundantly present in fruits, vegetables, grains, bark, roots, stems, flowers, tea, and wine [3]. In recent years, baicalein has been reported to possess antiviral activity against various viruses, including influenza virus, human immunodeficiency virus (HIV), hepatitis B virus (HBV), hepatitis C virus (HCV), and herpes simplex virus (HSV). In the case of HIV, baicalein has been found to target and inhibit the reverse transcriptase activity of the virus, inhibiting its replication [4,5]. Furthermore, baicalein and other flavonoids have been found to have potential inhibitory effects on the spike protein of SARS-CoV-2. Therefore, we found it worthwhile to design a dataset of virtual lead molecules and predict their binding profile as M^pro^ inhibitors [6]. We used molecular docking simulation technique that can be used to predict the interactions between ligands and M^pro^.

## 3 Data Description

Fig. 1.

**Fig. 1:**
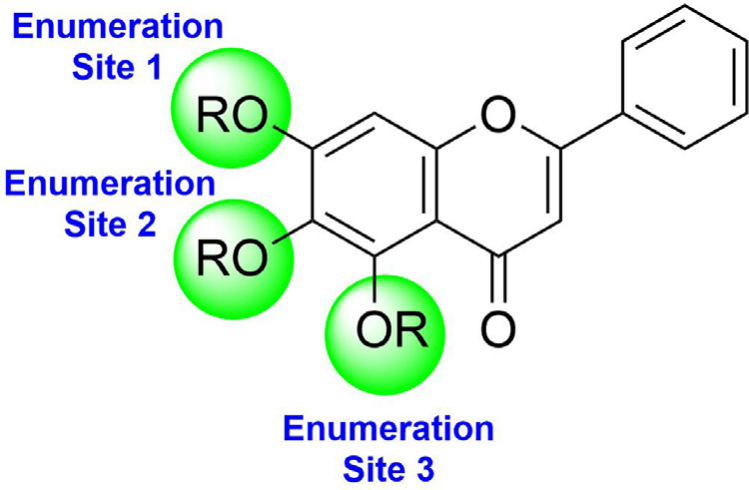
Chemical enumeration sites (1–3) on the baicalein scaffold selected for the design of baicalein analogues.

## 4 Experimental Design, Materials and Methods

### 4.1 Virtual screening procedure

Considering the limited approved drugs and the withdrawal of Remdesivir from clinical use for COVID-19, there is an essential need for targeted therapeutics and effective treatment options [7,8]. To address this issue, this study endeavours to utilise molecular docking based virtual screening tool to identify flavonoid hits as prospective anti-COVID-19 agents. This study aims to generate a reliable hypothetical compound that enables Medicinal Chemists to select for experimental studies. The study focuses on designing a library of molecules through structural modifications of the bioactive flavonoid “Baicalein.” The lead molecule would be optimised through structural modifications of the Baicalein to create a library of molecules. The subsequent step involves performing molecular docking based virtual screening of these designed ligands against the SARS-CoV-2 Main protease (M^pro^) to identify virtual hits for further study. Notably, no prior studies have been explored in this specific approach, highlighting the novelty and potential impact of this research. By studying the in-silico binding profile of these Baicalein analogues as M^pro^ inhibitors, valuable insights would contribute towards the development of potential therapeutic candidates against COVID-19.

### 4.2 Computational software applications

In these studies, we use the bare minimum of central hardware system configuration and run Schrödinger and other open-source computer-aided drug design software to perform molecular docking simulation-based virtual screening studies, which involve molecular modelling, energy minimisation, and some other docking procedures. For instance, Schrodinger software is used for molecular modelling, the energy minimisation of the ligand during ligand preparation, and the preparation and validation of proteins. likewise, grid files that represent the receptorʼs active site for ligand docking simulations in the process' later stages are generated using Schrödinger Software. Additionally, Schrödinger software is used for processes like molecular docking and visualisation of the docking results. The RCSB Protein Databank (https://www.rcsb.org/), ChemOffice 2020 (ChemDraw Professional, Chem3D), and other open-source computer-aided drug design software and websites are utilised as well. For instance, targeted protein selection is performed using RCSB Protein Databank (PDB ID: 6M2N), whereas the chemical structure of the ligand is drawn in two and three dimensions using ChemOffice 2020.

### 4.3 SARS-COV-2 main protease as targeted protein selection

The selected SARS-COV-2 main protease drug target from protein databank was retrieved based on the application of a set of filtering criteria that includes the three-dimensional structure determined using x-ray diffraction experiment, resolution between 1 and 3 Å, presence of co-crystalised ligand, no protein breaks in three-dimensional structure, Ramachandran plot statistics with desirable percentage of amino acids appeared in the most favourable region (>90 %) and, disallowed region (0 %) respectively. In addition to the above filters, we also applied a specific requirement that is presence of flavonoid with the experimentally determined SARS-COV-2 main protease inhibitory property. The above systematic procedure resulted in a protein drug target with PDB ID: 6M2N, in which Baicalein flavonoid is the co-crystalised ligand. This PDB target was selected to perform molecular docking simulation-based virtual screening.

### 4.4 Ligand design using Schrödinger software and maestro

The ligand database is created using Schrodinger Softwareʼs Maestro R-group enumeration tool, which is based on the co-crystallised bioactive ligand conformation of “Baicalein.” Afterward, on the Baicalein scaffold, 2098 R-Groups from the eMolecules library are substituted into three different substitution sites (Fig. 1). The prepared compounds will then be subjected to energy minimisation using the LigPrep module in the Schrodinger software, as energy minimisation can prevent any steric clashes or the occurrence of inappropriate geometrical structure on the ligandʼs surface. Energy minimisation, for instance, can clear the surface of the ligand of any unwanted hydrogen atoms or overlapping atoms. Because a molecule's potential energy varies depending on its components, it is critical to run an energy minimisation procedure to reduce this energy and search for the stable conformer that is structurally closest to the starting molecular arrangement. As a result, it enables us to explicitly consider either partial or rigidity of the ligand and receptor when defining the conformational space, only then should these energy-minimised structures be considered for molecular docking simulation-based virtual screening study [9]. At the end of ligand preparation, a total number of 2563 Baicalein analogues are generated for each enumeration site respectively.

### 4.5 Molecular docking simulation-based virtual screening procedure

**Fig. 2:**
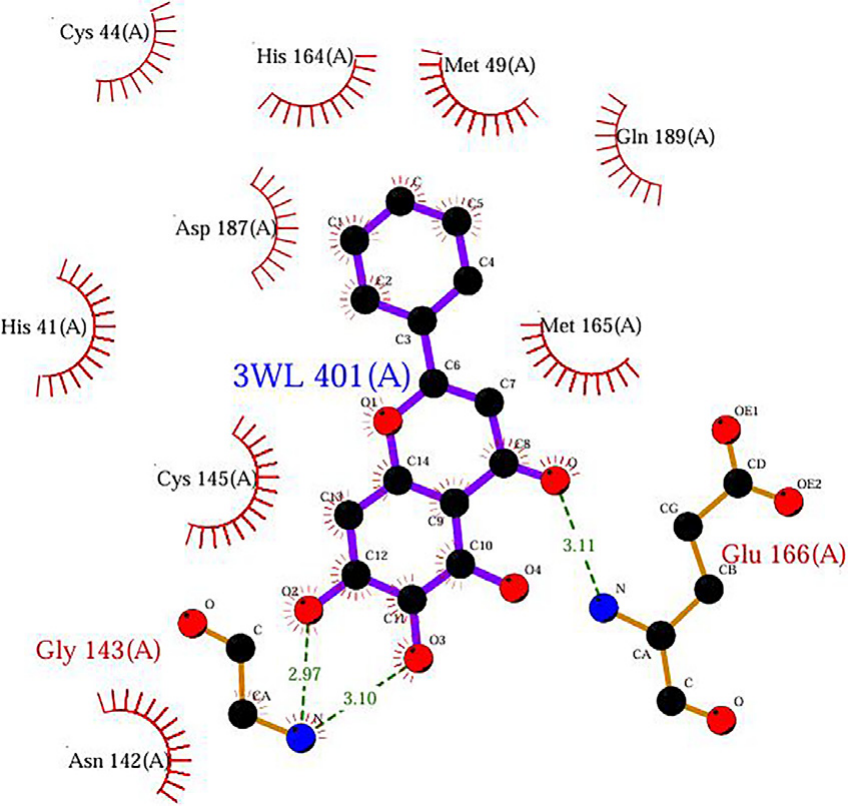
Binding interaction diagram of the co-crystallized ligand baicalein at the SARS-CoV-2 M^pro^ target's binding site region (PDB ID: 6M2N).

In the present study, molecular docking simulations-based virtual screening procedures are performed on the energy-minimised ligand, which we prepared using Maestro R-group enumeration tool and the selected target protein, which is SARS-COV-2 Main Protease (PDB: 6M2N) co-crystallized with baicalein (Fig. 2). Through different docking filters including High-throughput Virtual Screening (HTVS), Standard Precision (SP), and Extra Precision (XP), molecular docking protocols can facilitate virtual hit identification of prepared ligand against the selected target protein by using Schrodinger Software (Glide). The “hit molecules” obtained from the molecular docking procedure will be further analysed for their binding properties including binding mode, binding interactions, and binding energy of putative stable ligand conformations using Maestro. In addition, a standard Baicalein structure is also prepared and acts as a target for comparison between baicalein and its analogue.

### 4.6 Visualisation of results using maestro and Microsoft Excel

Maestro is a structural visualisation tool included with Schrodinger Software. The outcomes presented using different parameters that include ligand-binding energies, ligand binding interactions, and ligand orientation diagrams. Maestro allows visualisation and for comparing results, which increases the insight and understanding potential of the results obtained. The visualisation of ligand-binding interactions provides additional information, such as repeated amino acids that appear in various rankings and docking methods (HTVS, SP, XP). Additionally, the enhanced diagram of the binding interactions between the ligand and the targeted binding site is displayed by ligand-binding site interactions. The ligand orientation diagram also illustrates the differences in ligand orientation and chemical structure between Baicalein and its analogues. Furthermore, Microsoft Excel is used to perform descriptive statistical data analysis and interpret the current study's results.

### 4.7 Method validation

**Fig. 3:**
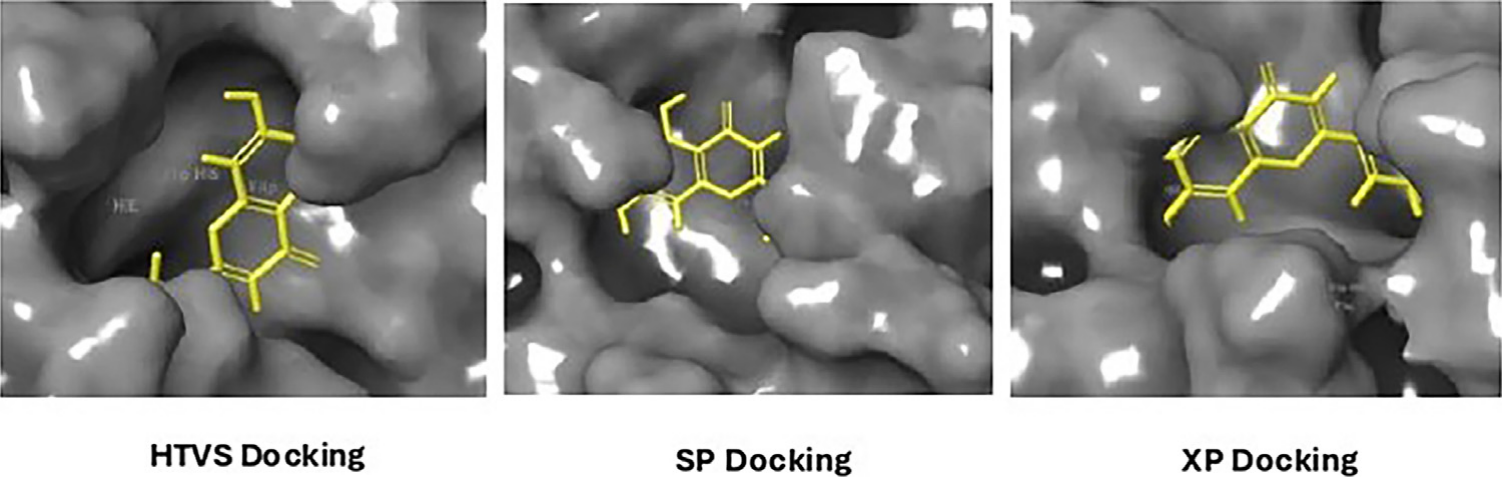
Binding orientation of the baicalein docked using HTVS, SP, and XP methods at the SARS-CoV-2 M^pro^ target's binding site region (PDB ID: 6M2N).

**Fig. 4:**
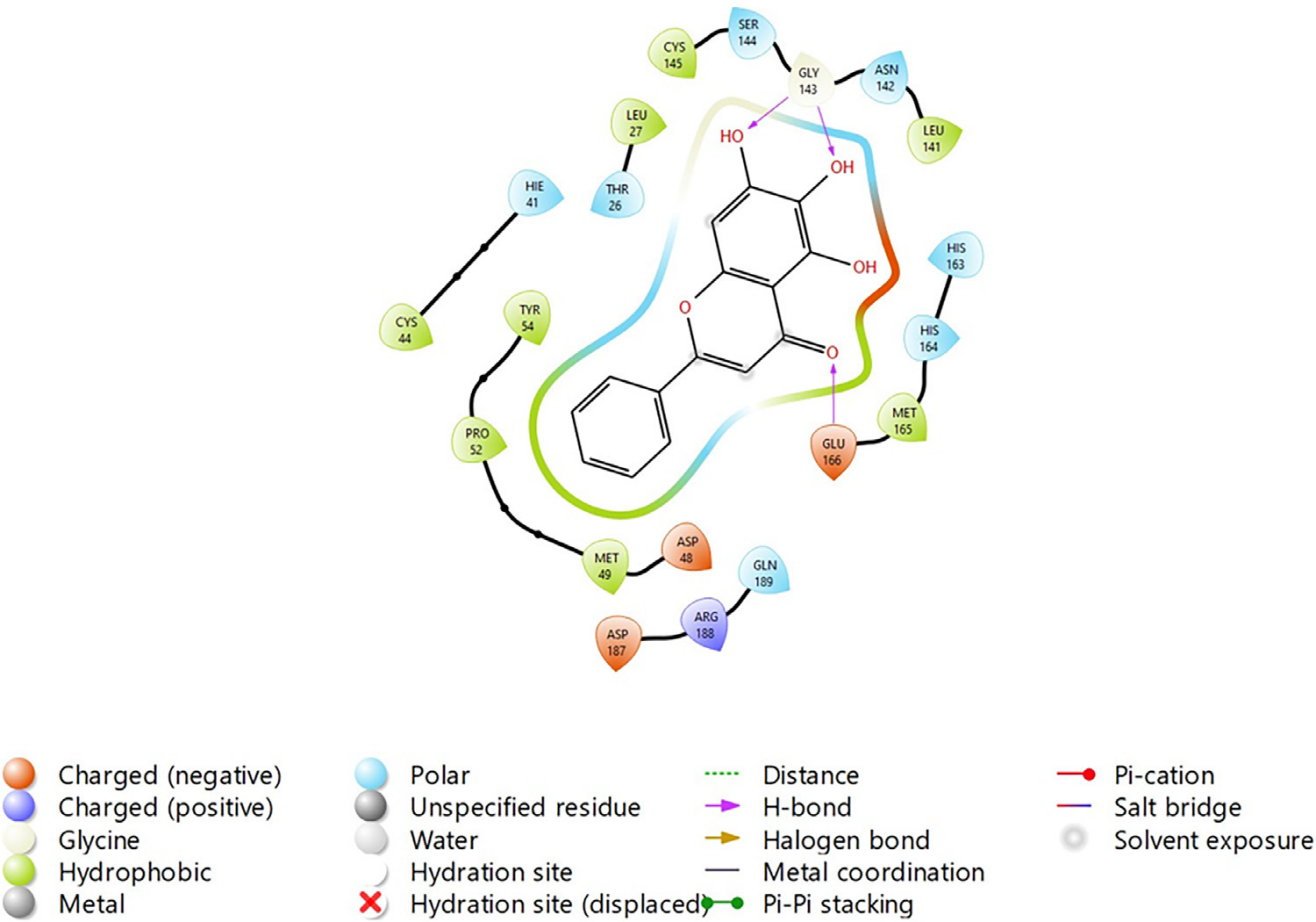
Binding interactions diagram of the baicalein docked using HTVS method at the SARS-CoV-2 M^pro^ target's binding site region (PDB ID: 6M2N).

**Fig. 5:**
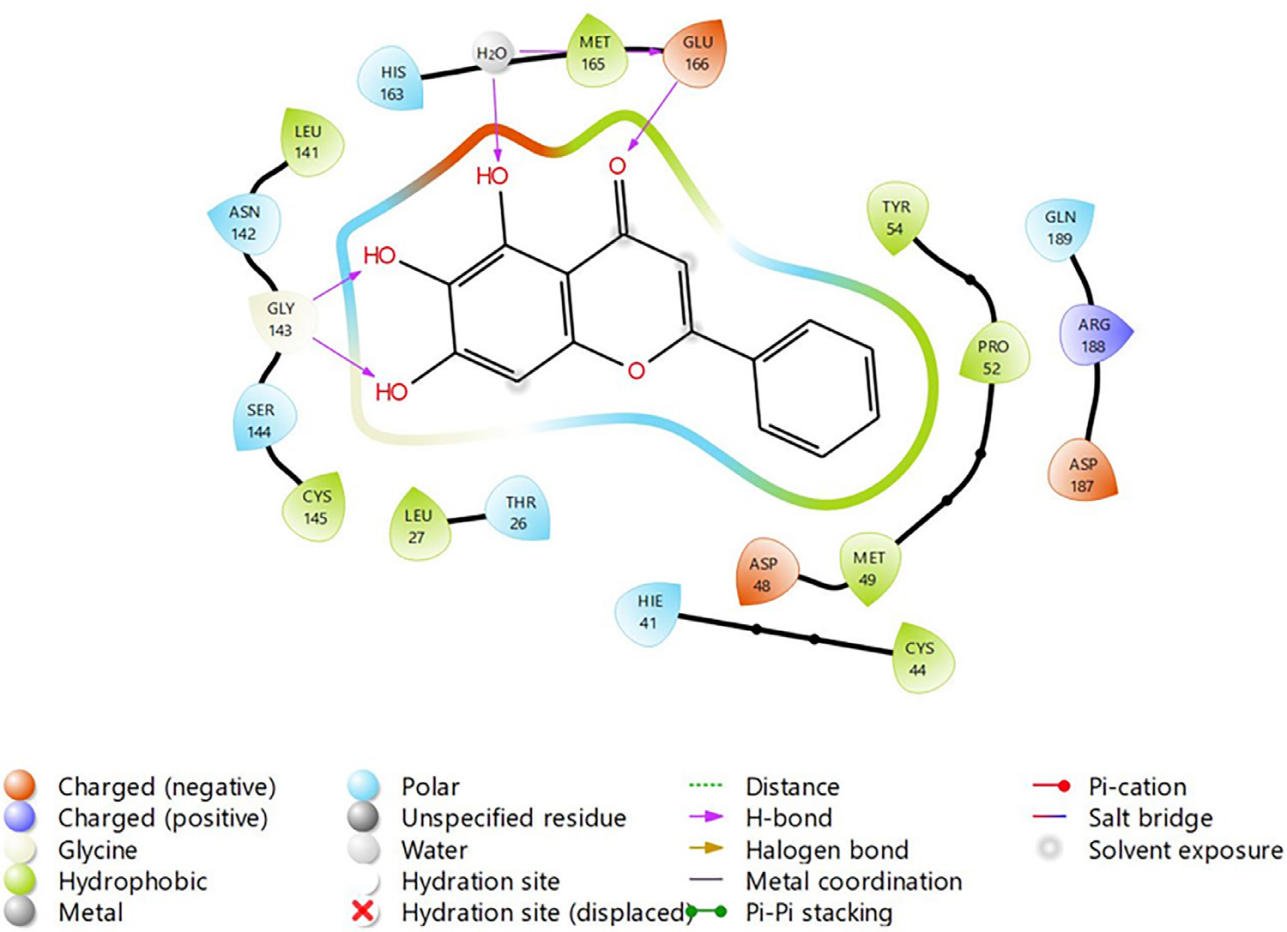
Binding interactions diagram of the baicalein docked using SP method at the SARS-CoV-2 M^pro^ target's binding site region (PDB ID: 6M2N).

**Fig. 6:**
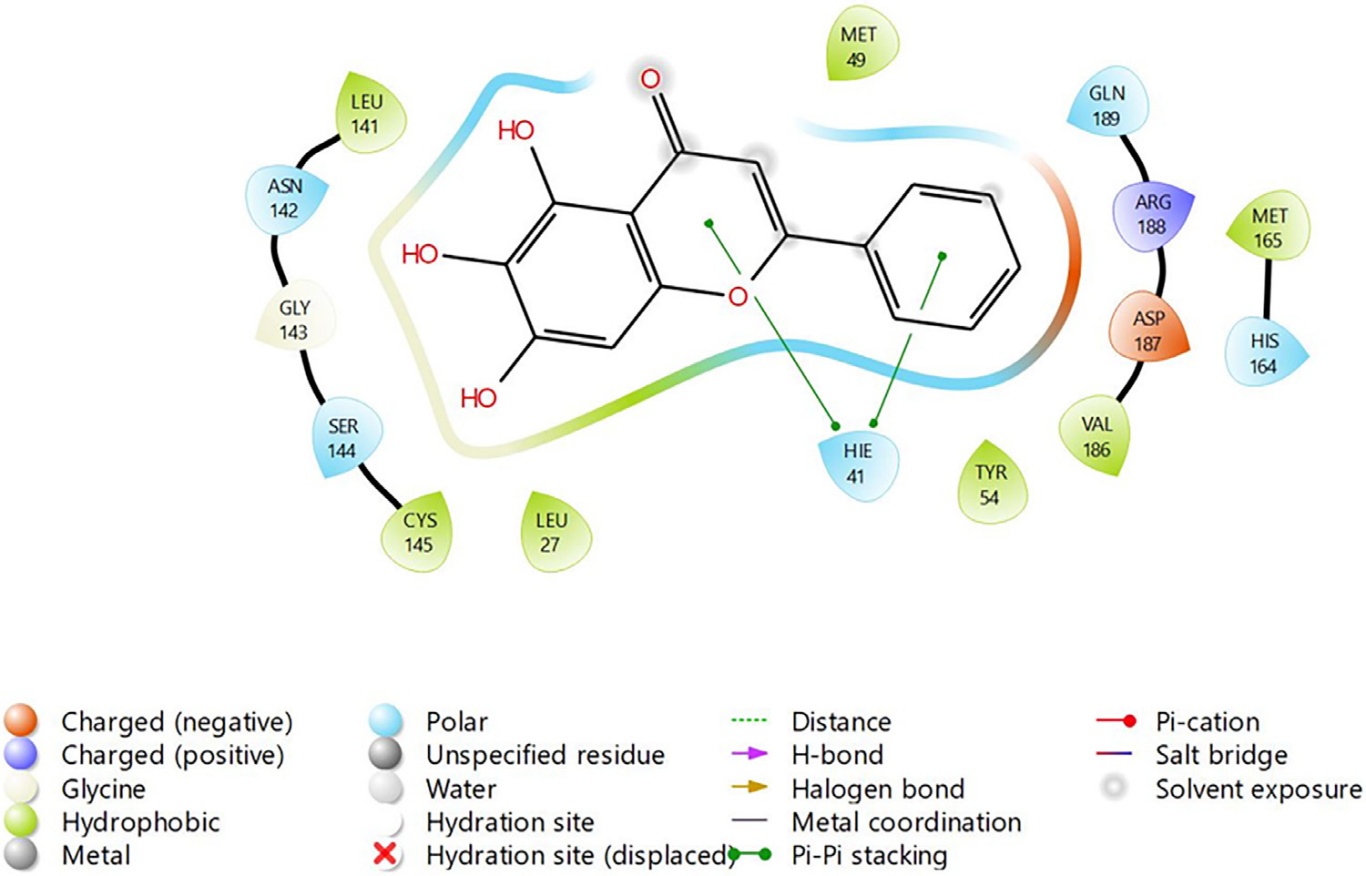
Binding interactions diagram of the baicalein docked using XP method at the SARS-CoV-2 M^pro^ target's binding site region (PDB ID: 6M2N).

**Fig. 7:**
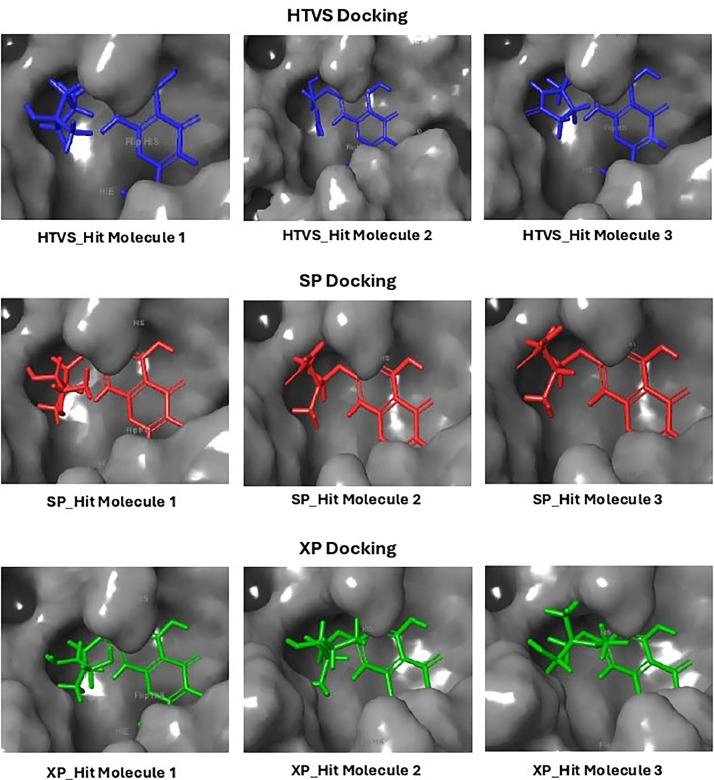
Binding orientation of the top three stable virtual hit molecules identified from HTVS, SP, and XP docking methods at the SARS-CoV-2 M^pro^ target's binding site region (PDB ID: 6M2N), the substituted baicalein analogues designed based on the chemical enumeration of site 1.

**Fig. 8:**
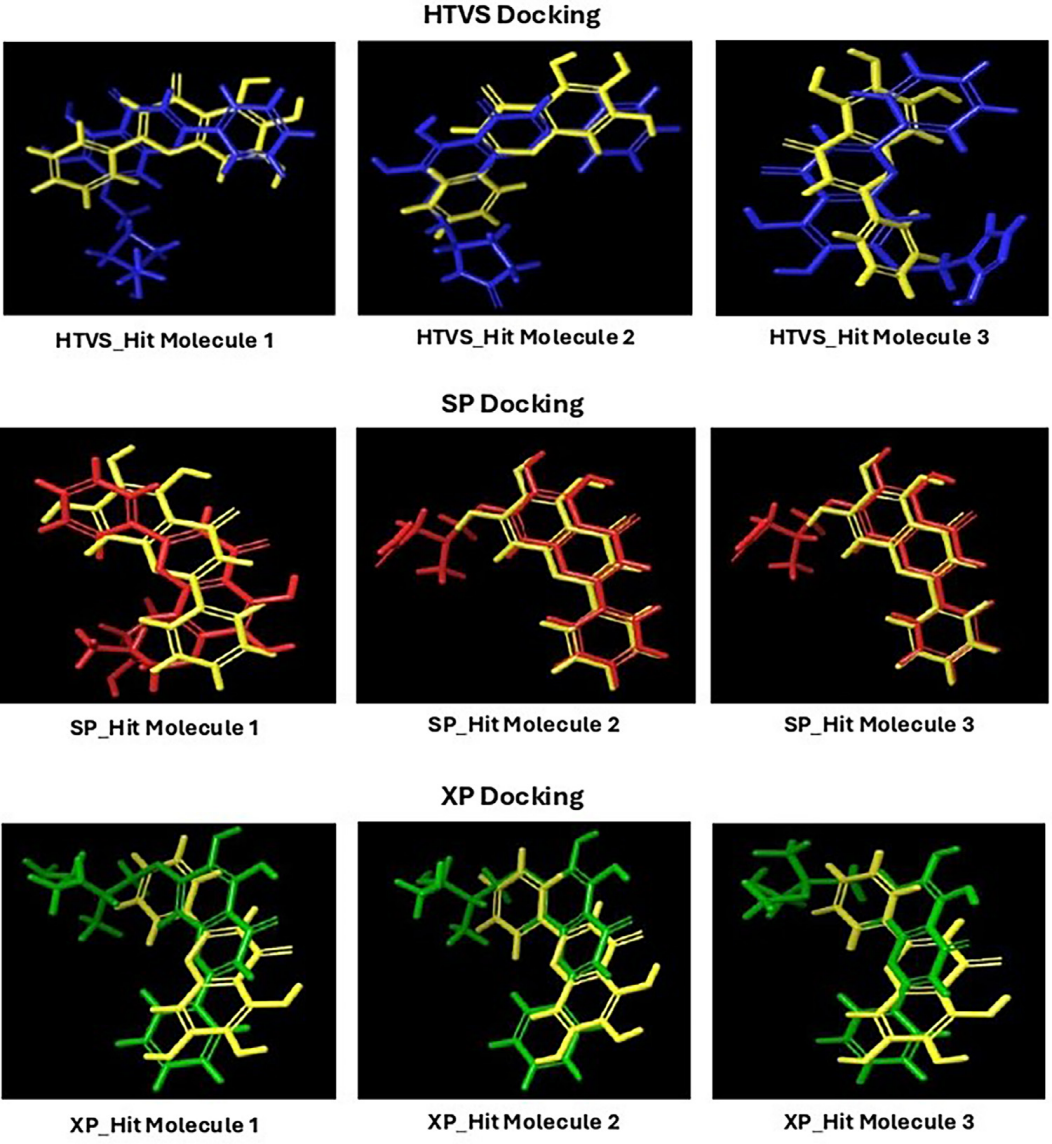
Superimposed stable binding conformations of the baicalein and the top three stable virtual hit molecules identified from HTVS, SP, and XP docking methods at the SARS-CoV-2 M^pro^ target's binding site region (PDB ID: 6M2N), the substituted baicalein analogues designed based on the chemical enumeration of site 1.

**Fig. 9:**
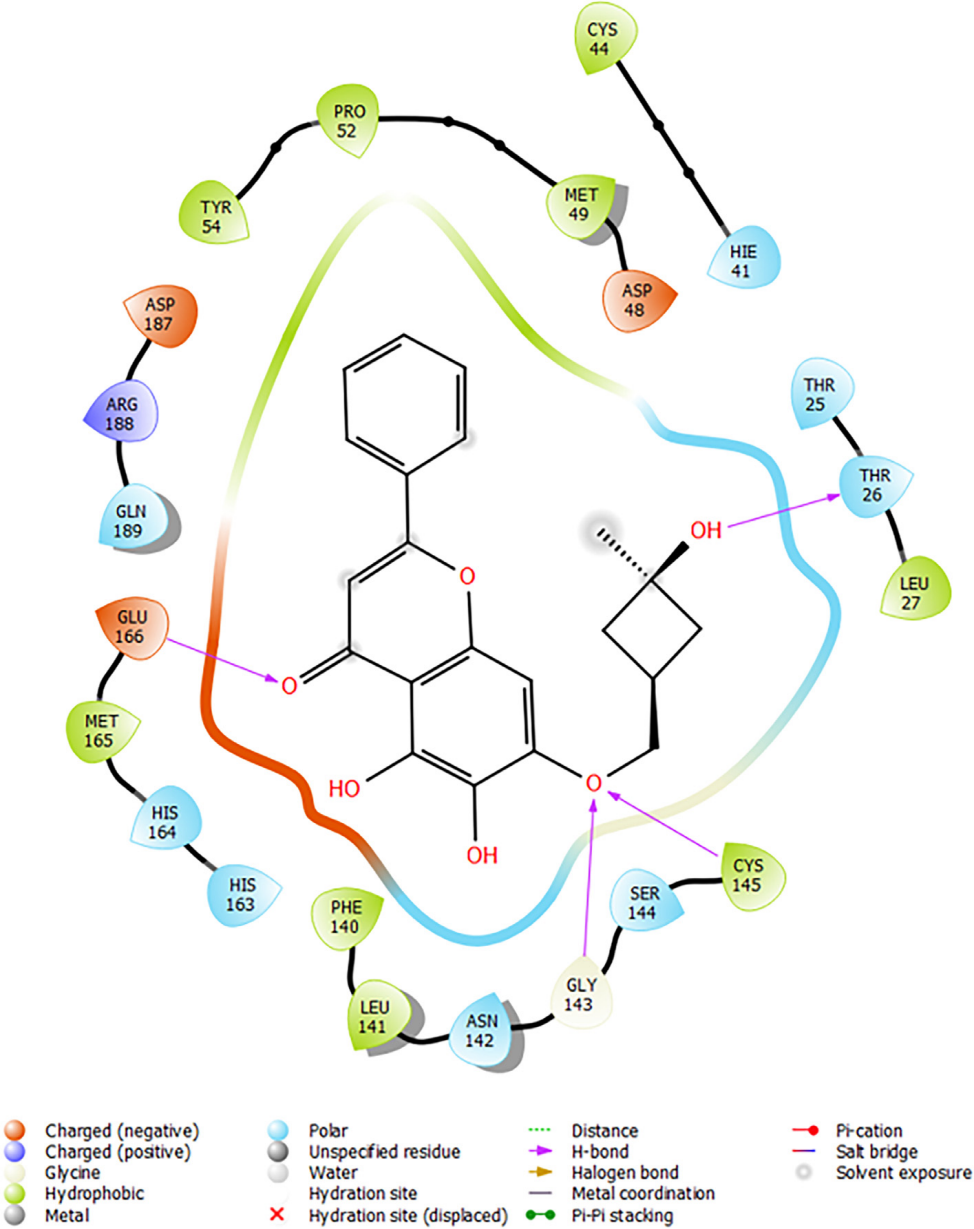
Binding interactions diagram of the top three stable virtual hit molecule 1 identified from the HTVS docking method at the SARS-CoV-2 M^pro^ target's binding site region (PDB ID: 6M2N), the substituted baicalein analogues designed based on the chemical enumeration of site 1.

**Fig. 10:**
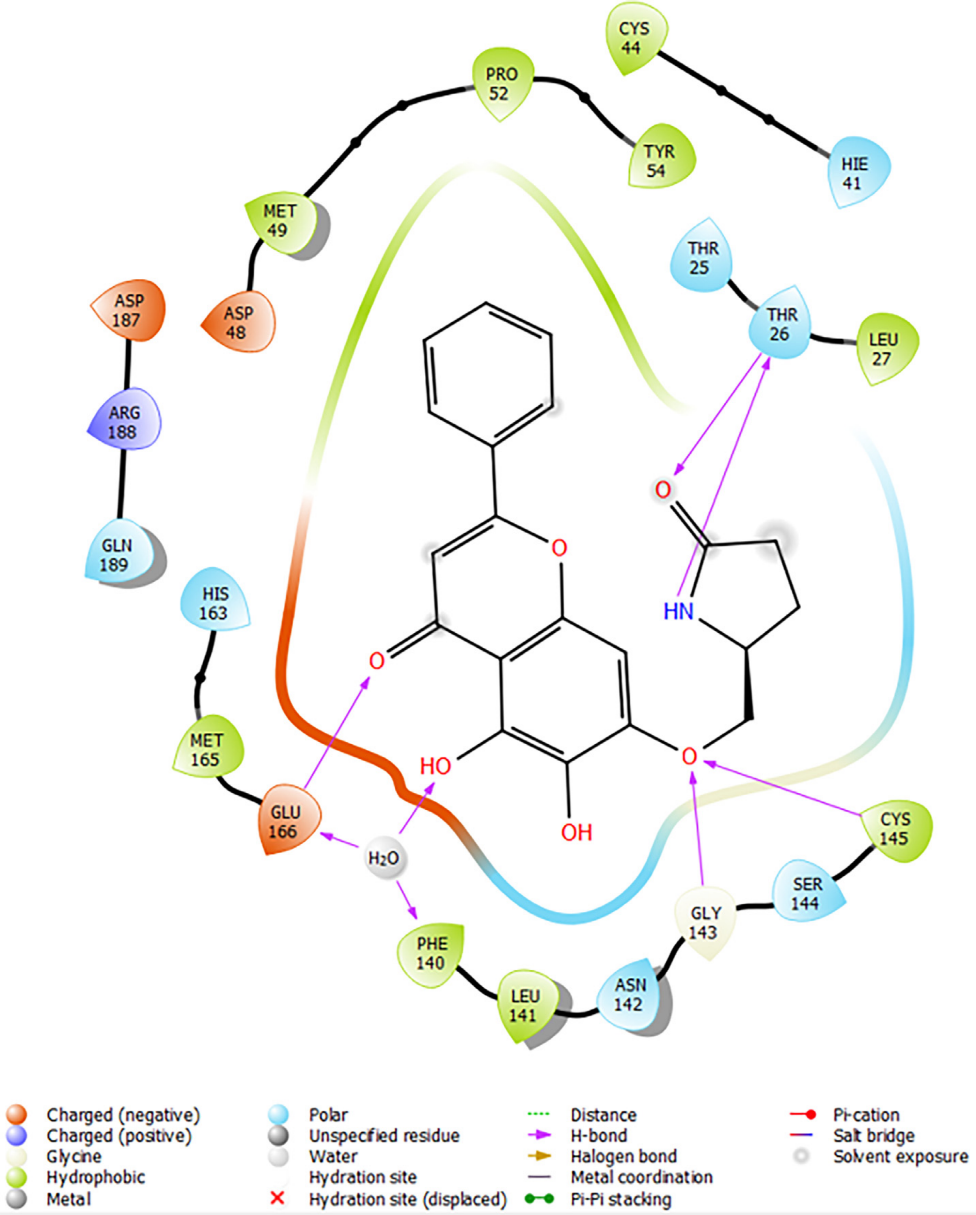
Binding interactions diagram of the top three stable virtual hit molecule 2 identified from the HTVS docking method at the SARS-CoV-2 M^pro^ target's binding site region (PDB ID: 6M2N), the substituted baicalein analogues designed based on the chemical enumeration of site 1.

**Fig. 11:**
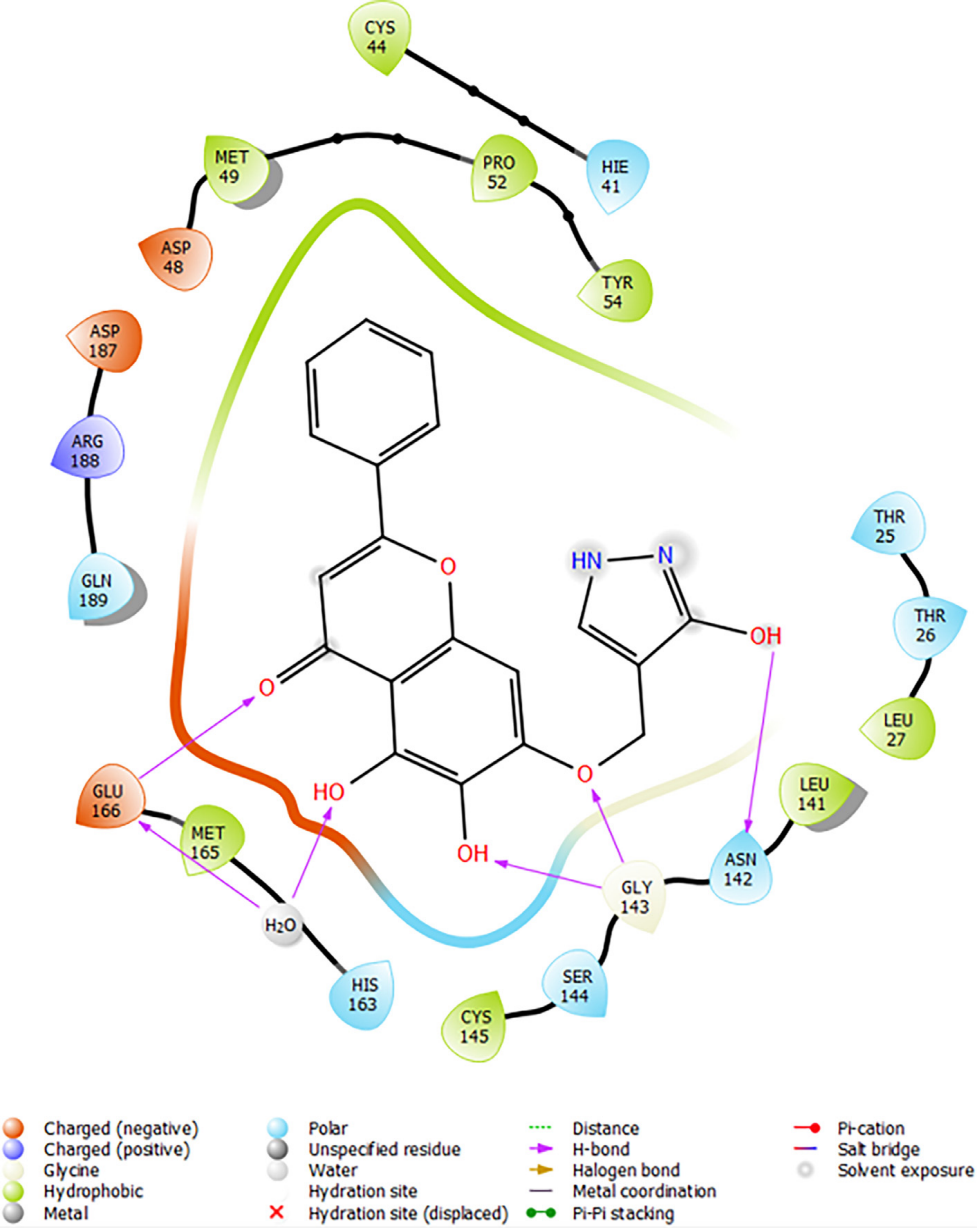
Binding interactions diagram of the top three stable virtual hit molecule 3 identified from the HTVS docking method at the SARS-CoV-2 M^pro^ targetʼs binding site region (PDB ID: 6M2N), the substituted baicalein analogues designed based on the chemical enumeration of site 1.

**Fig. 12:**
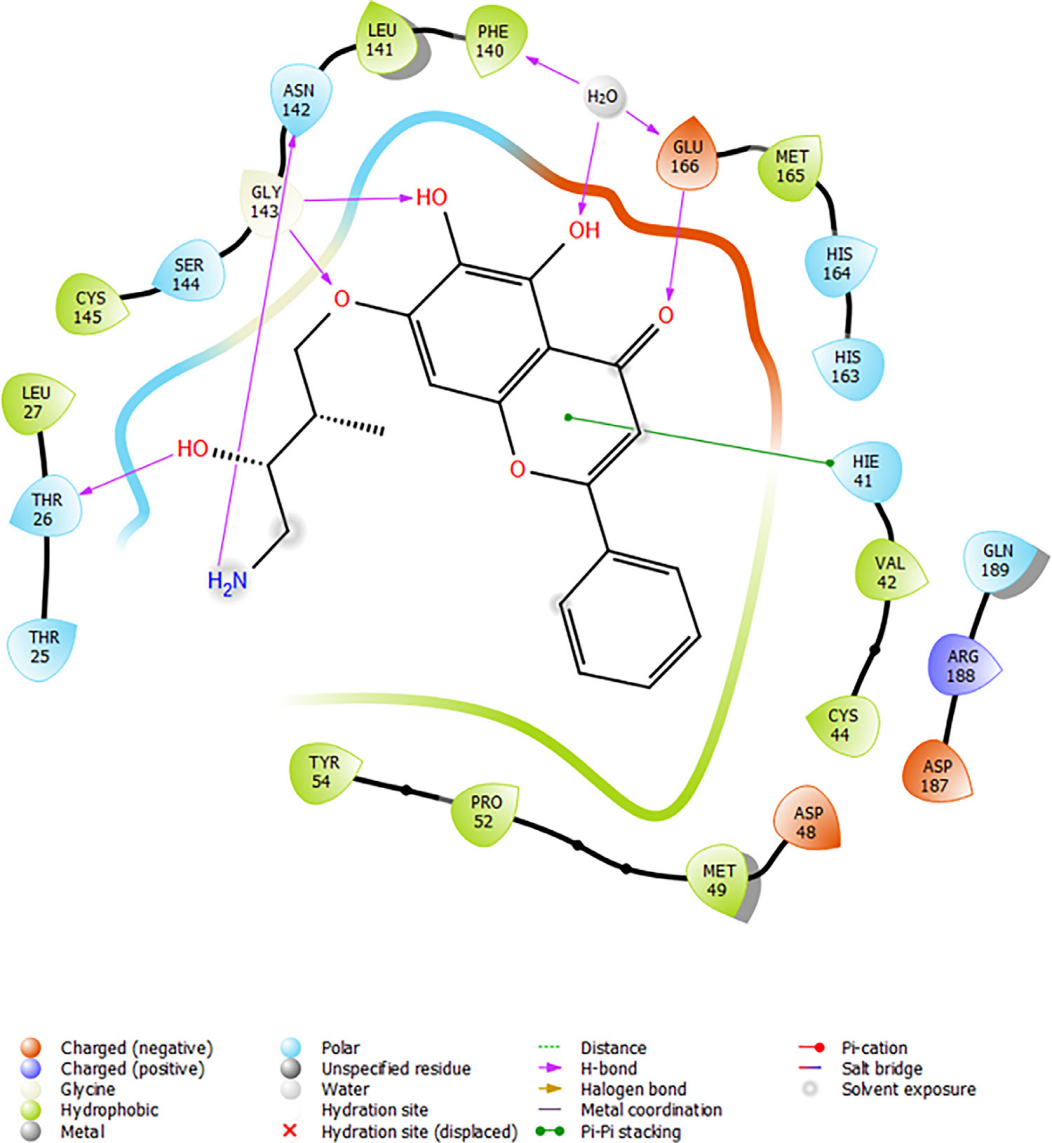
Binding interactions diagram of the top three stable virtual hit molecule 1 identified from the SP docking method at the SARS-CoV-2 M^pro^ target's binding site region (PDB ID: 6M2N), the substituted baicalein analogues designed based on the chemical enumeration of site 1.

**Fig. 13:**
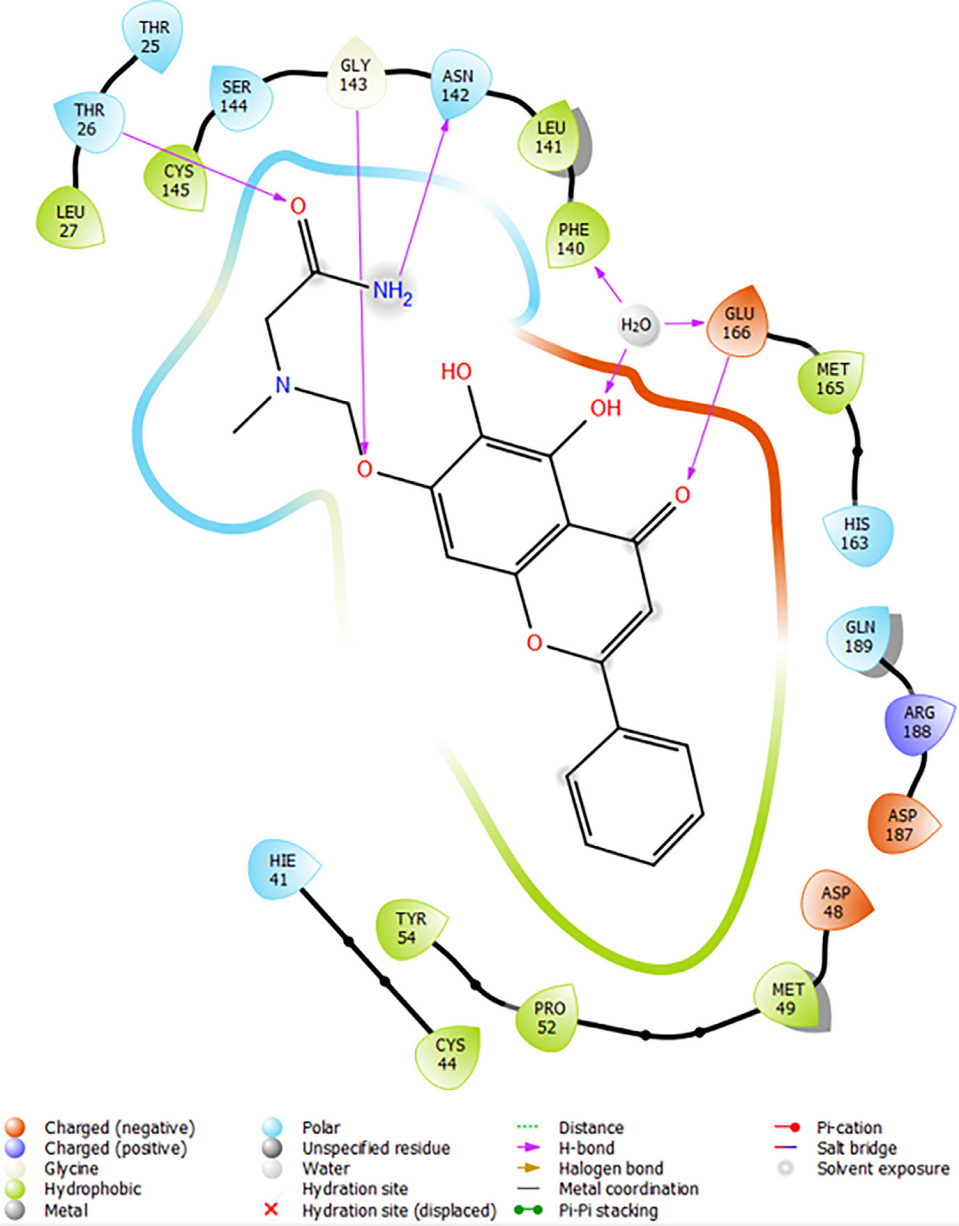
Binding interactions diagram of the top three stable virtual hit molecule 2 identified from the SP docking method at the SARS-CoV-2 M^pro^ target's binding site region (PDB ID: 6M2N), the substituted baicalein analogues designed based on the chemical enumeration of site 1.

**Fig. 14:**
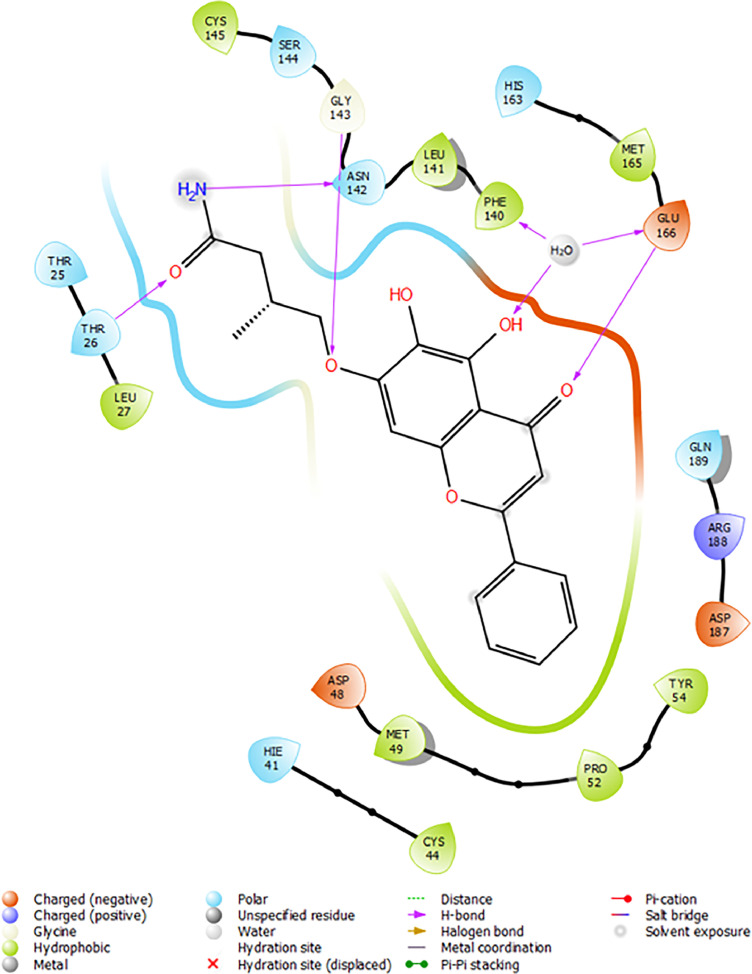
Binding interactions diagram of the top three stable virtual hit molecule 3 identified from the SP docking method at the SARS-CoV-2 M^pro^ target's binding site region (PDB ID: 6M2N), the substituted baicalein analogues designed based on the chemical enumeration of site 1.

**Fig. 15:**
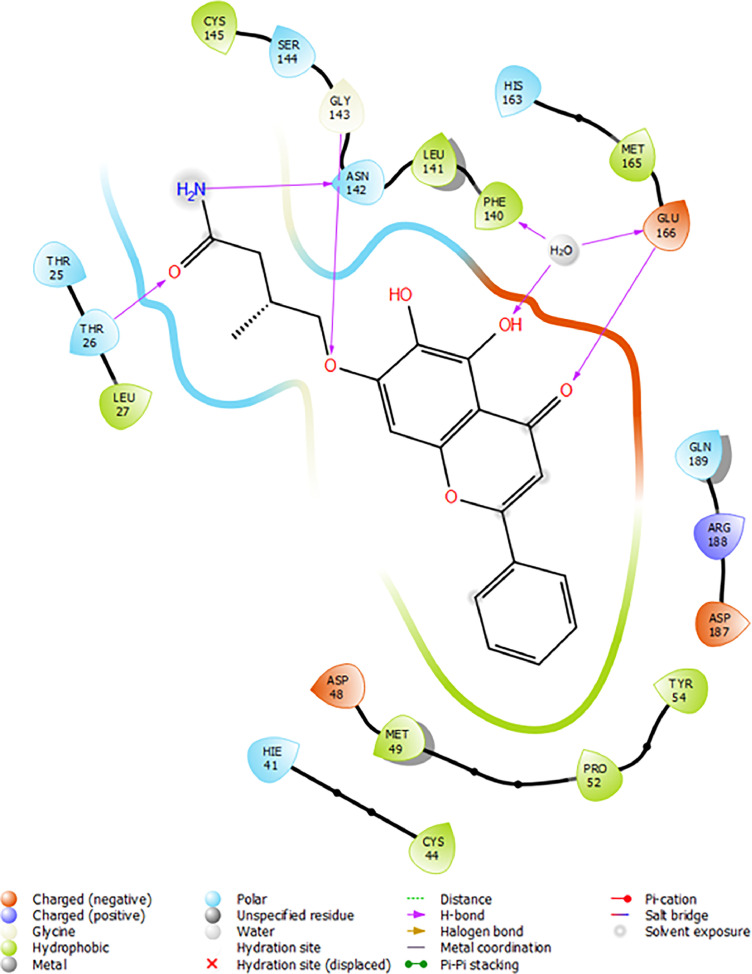
Binding interactions diagram of the top three stable virtual hit molecule 1 identified from the XP docking method at the SARS-CoV-2 M^pro^ target's binding site region (PDB ID: 6M2N), the substituted baicalein analogues designed based on the chemical enumeration of site 1.

**Fig. 16:**
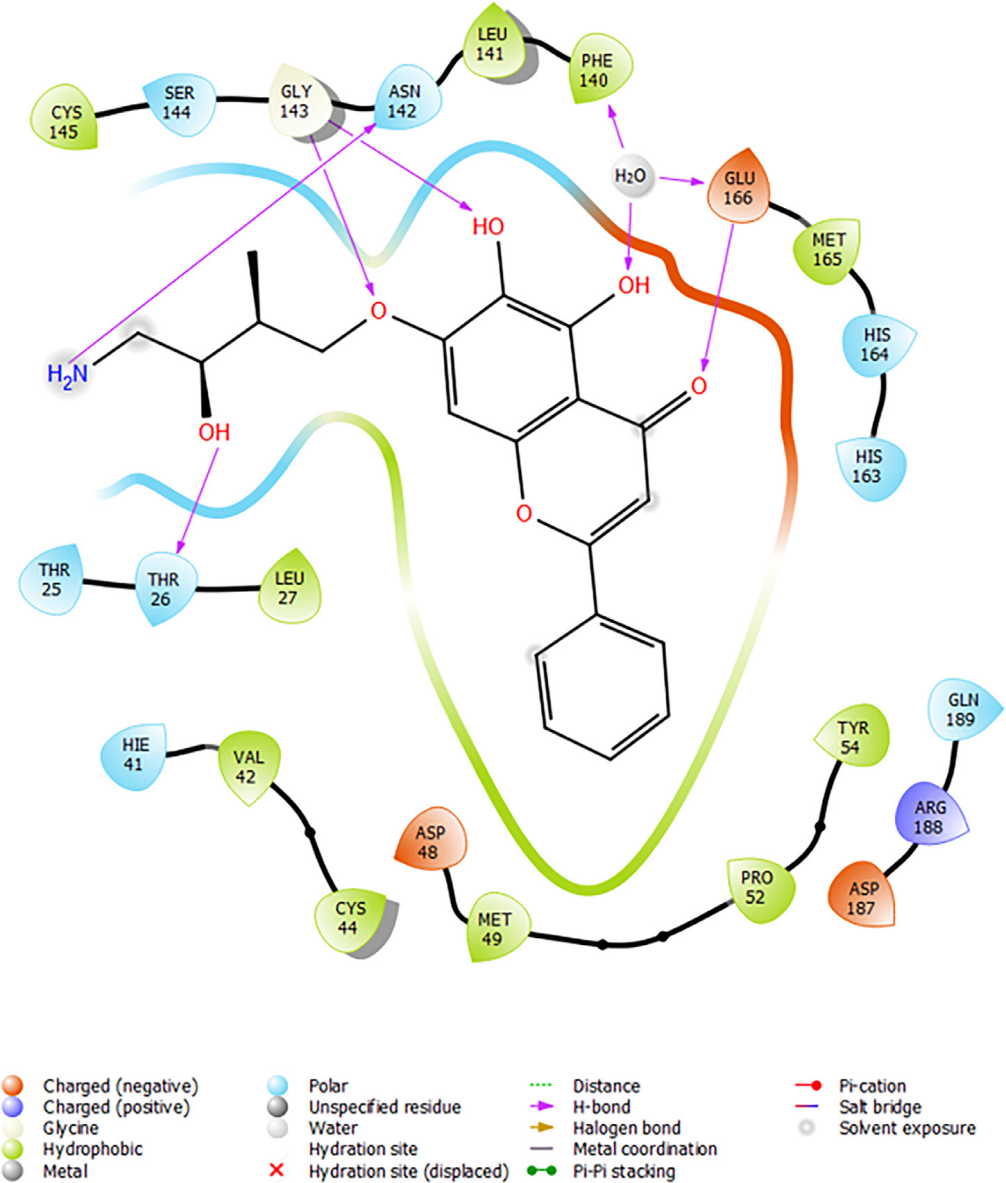
Binding interactions diagram of the top three stable virtual hit molecule 2 identified from the XP docking method at the SARS-CoV-2 M^pro^ targetʼs binding site region (PDB ID: 6M2N), the substituted baicalein analogues designed based on the chemical enumeration of site 1.

**Fig. 17:**
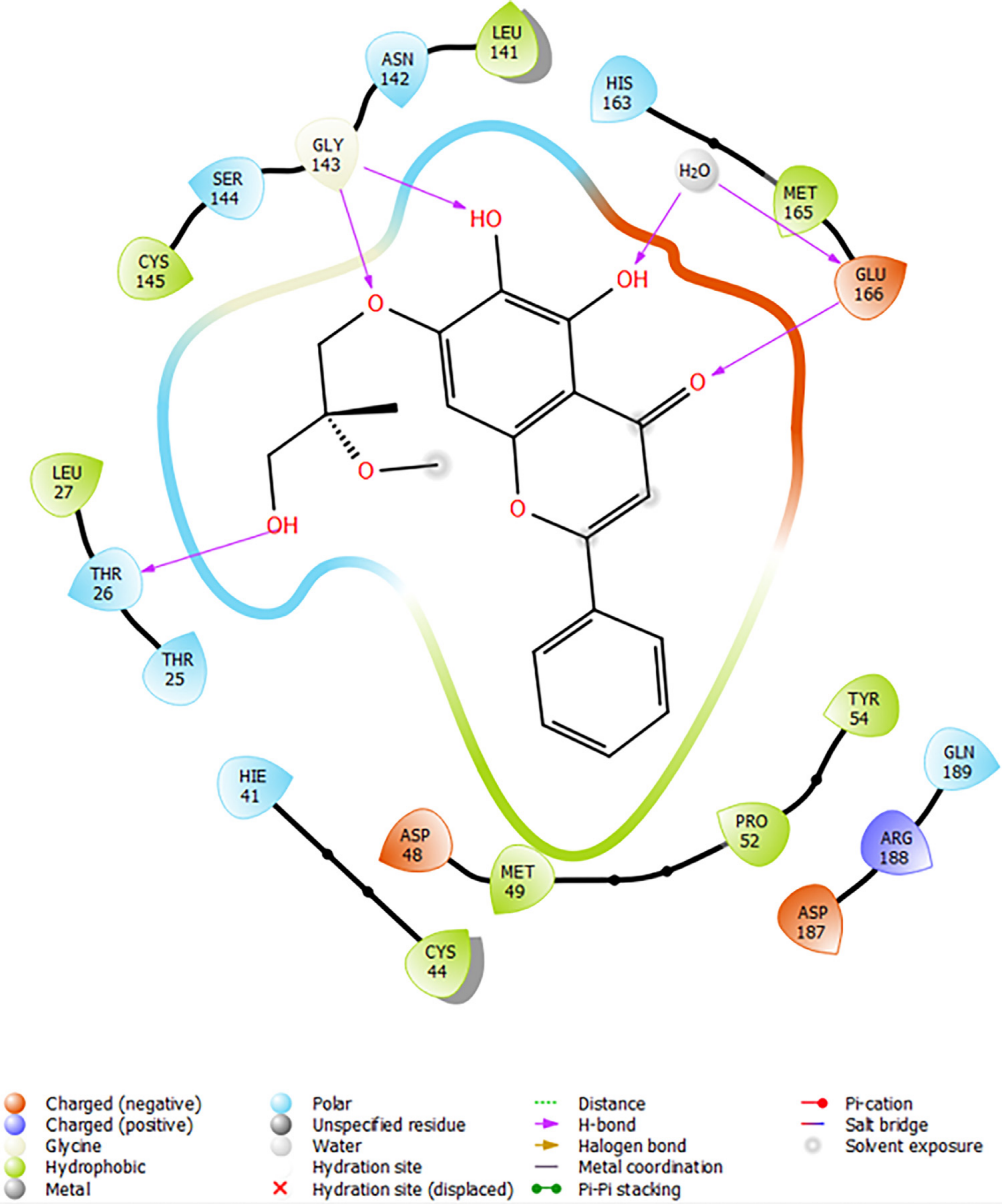
Binding interactions diagram of the top three stable virtual hit molecule 3 identified from the XP docking method at the SARS-CoV-2 M^pro^ targetʼs binding site region (PDB ID: 6M2N), the substituted baicalein analogues designed based on the chemical enumeration of site 1.

**Fig. 18:**
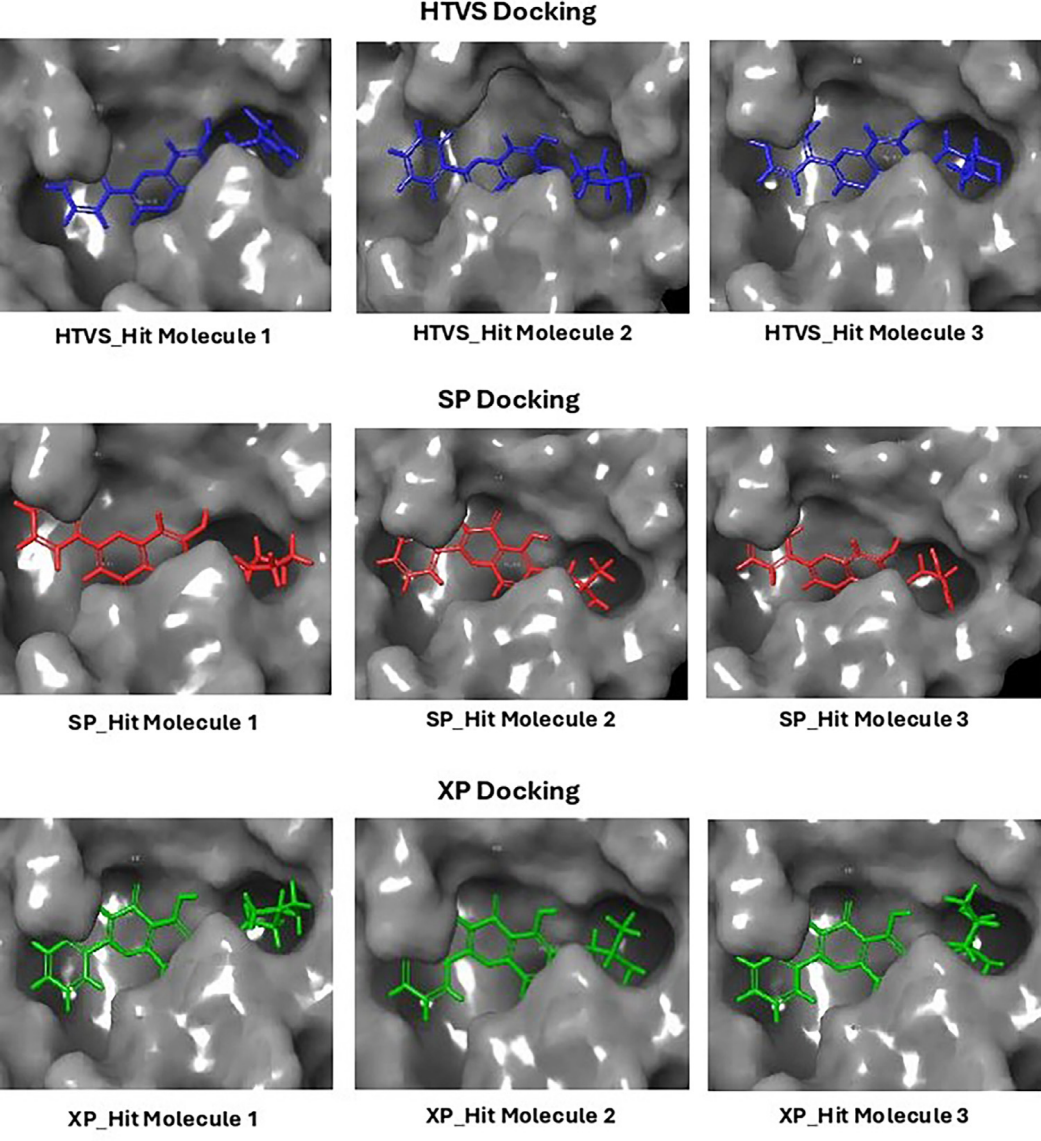
Binding orientation of the top three stable virtual hit molecules identified from HTVS, SP, and XP docking methods at the SARS-CoV-2 M^pro^ target's binding site region (PDB ID: 6M2N), the substituted baicalein analogues designed based on the chemical enumeration of site 2.

**Fig. 19:**
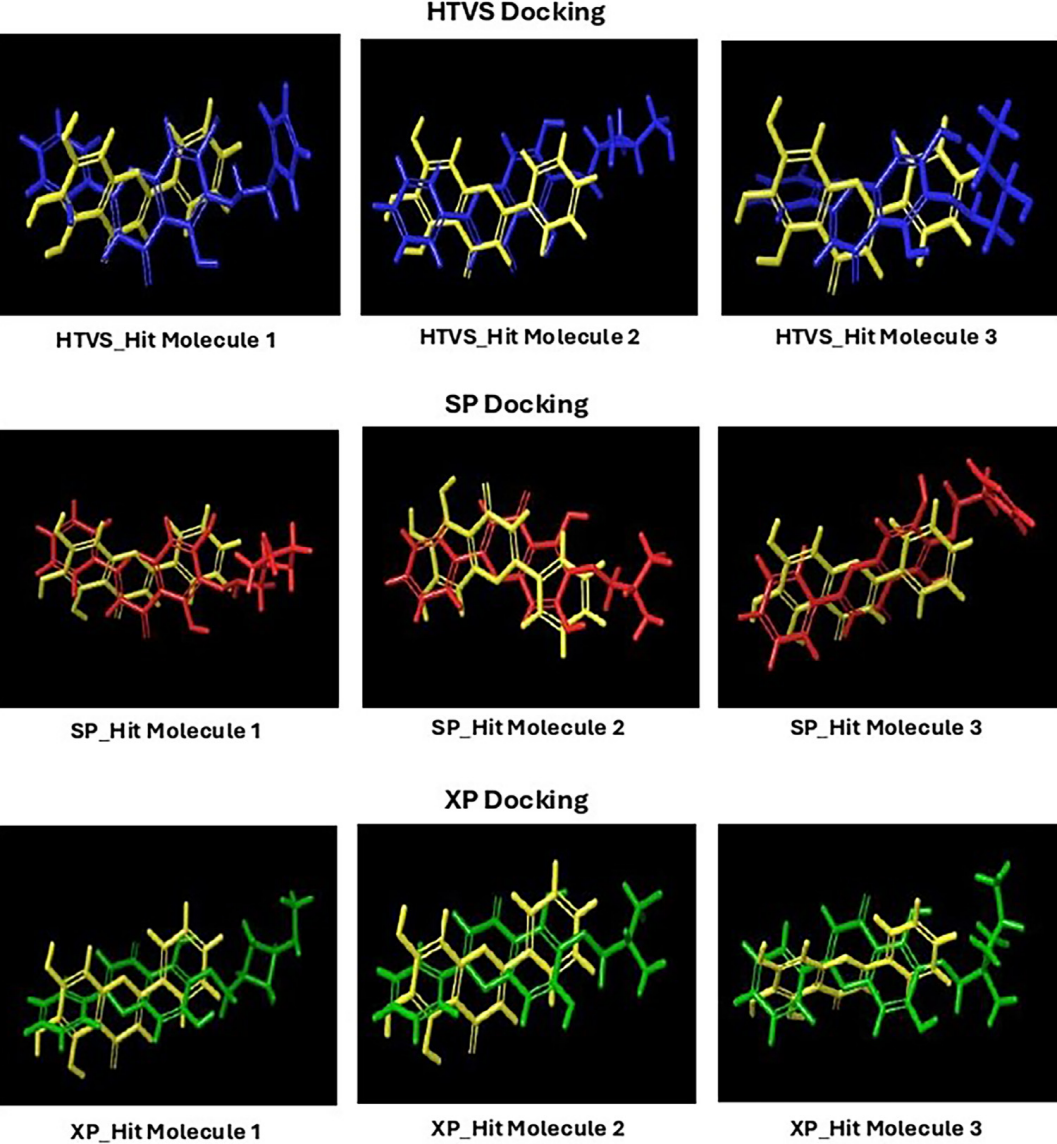
Superimposed stable binding conformations of the baicalein and the top three stable virtual hit molecules identified from HTVS, SP, and XP docking methods at the SARS-CoV-2 M^pro^ targetʼs binding site region (PDB ID: 6M2N), the substituted baicalein analogues designed based on the chemical enumeration of site 2.

**Fig. 20:**
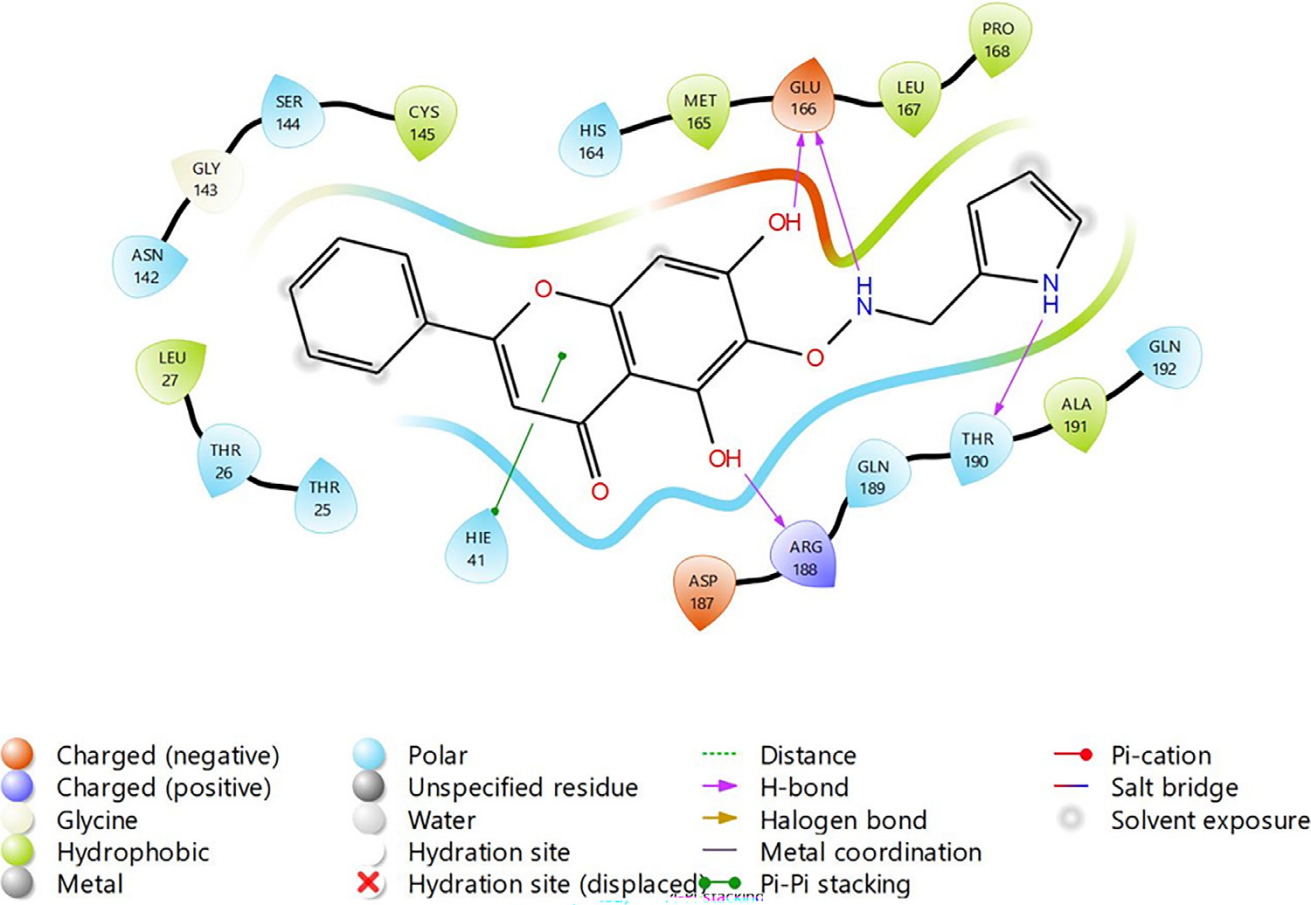
Binding interactions diagram of the top three stable virtual hit molecule 1 identified from the HTVS docking method at the SARS-CoV-2 M^pro^ targetʼs binding site region (PDB ID: 6M2N), the substituted baicalein analogues designed based on the chemical enumeration of site 2.

**Fig. 21:**
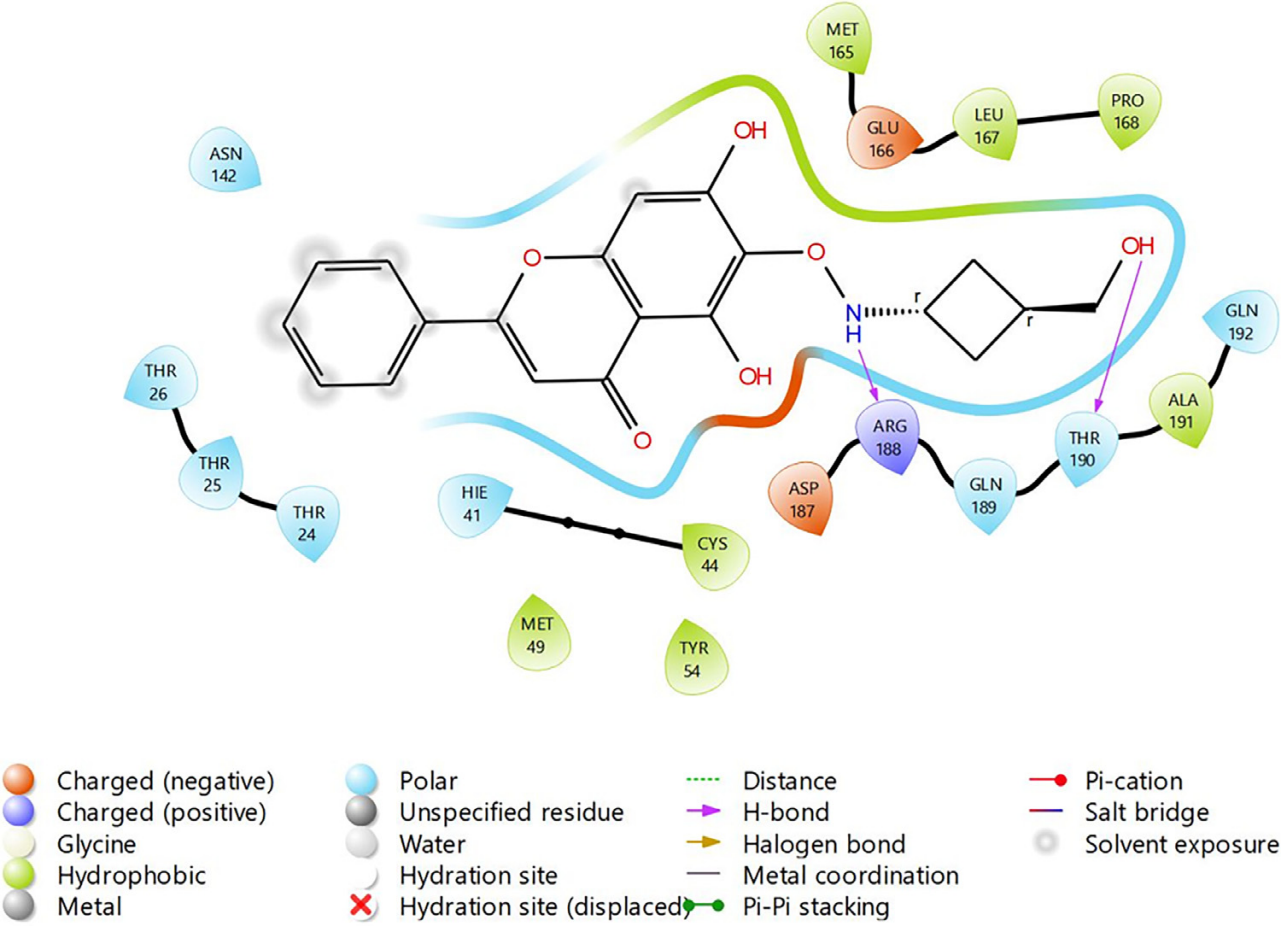
Binding interactions diagram of the top three stable virtual hit molecule 2 identified from the HTVS docking method at the SARS-CoV-2 M^pro^ targetʼs binding site region (PDB ID: 6M2N), the substituted baicalein analogues designed based on the chemical enumeration of site 2.

**Fig. 22:**
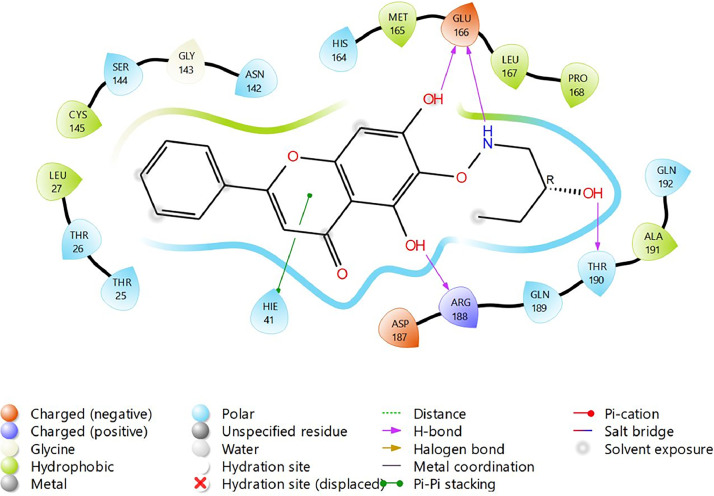
Binding interactions diagram of the top three stable virtual hit molecule 3 identified from the HTVS docking method at the SARS-CoV-2 M^pro^ targetʼs binding site region (PDB ID: 6M2N), the substituted baicalein analogues designed based on the chemical enumeration of site 2.

**Fig. 23:**
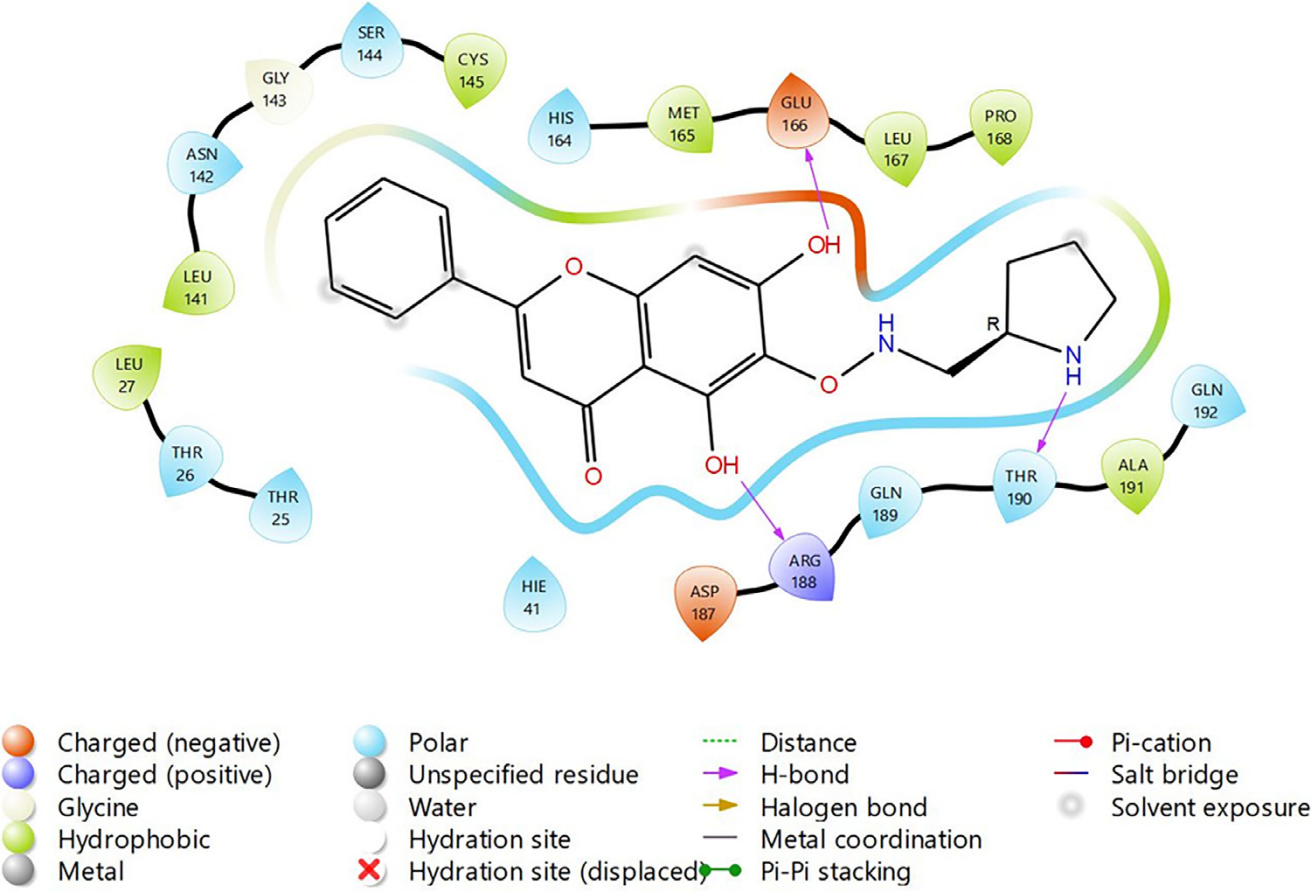
Binding interactions diagram of the top three stable virtual hit molecule 1 identified from the SP docking method at the SARS-CoV-2 M^pro^ targetʼs binding site region (PDB ID: 6M2N), the substituted baicalein analogues designed based on the chemical enumeration of site 2.

**Fig. 24:**
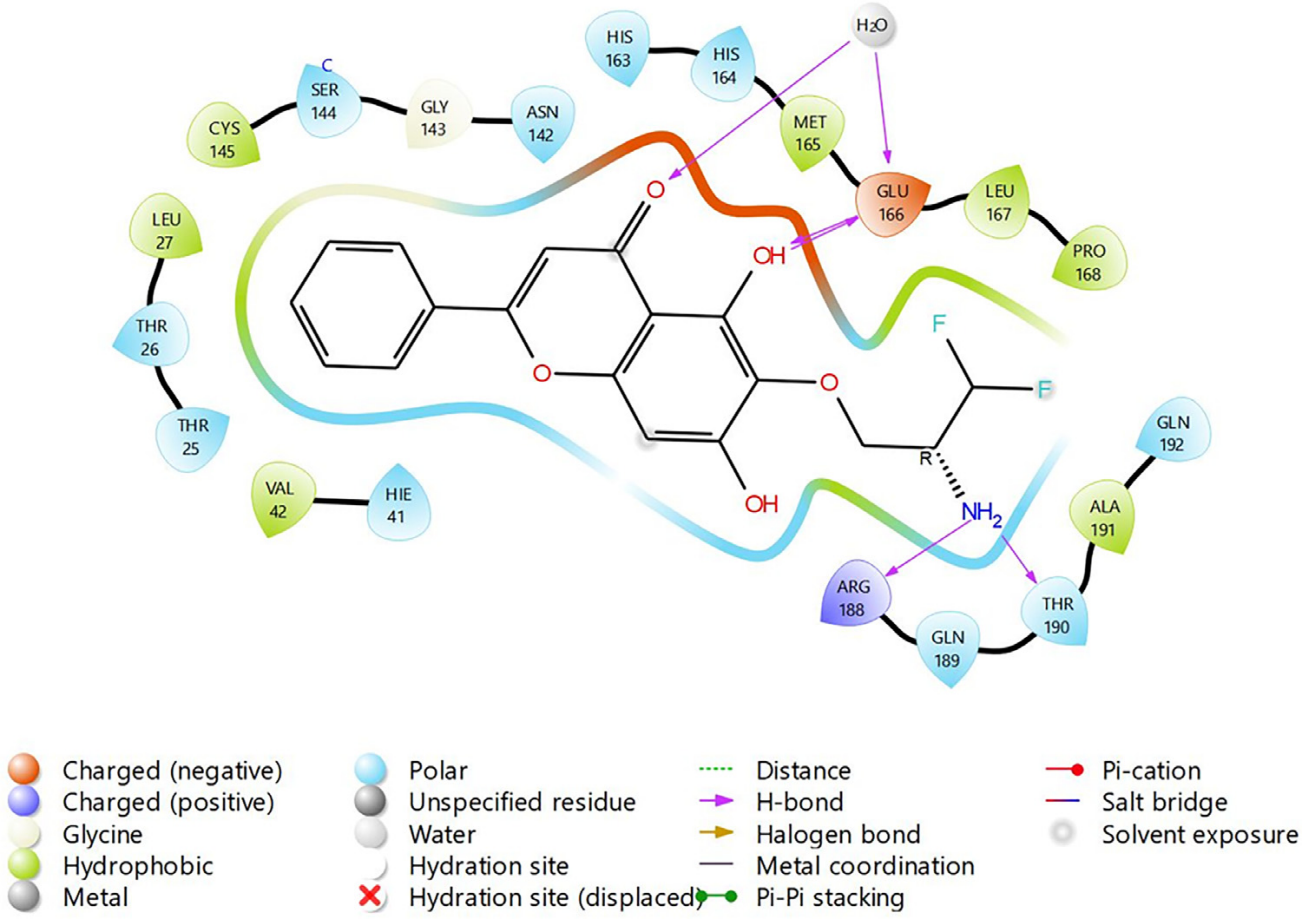
Binding interactions diagram of the top three stable virtual hit molecule 2 identified from the SP docking method at the SARS-CoV-2 M^pro^ targetʼs binding site region (PDB ID: 6M2N), the substituted baicalein analogues designed based on the chemical enumeration of site 2.

**Fig. 25:**
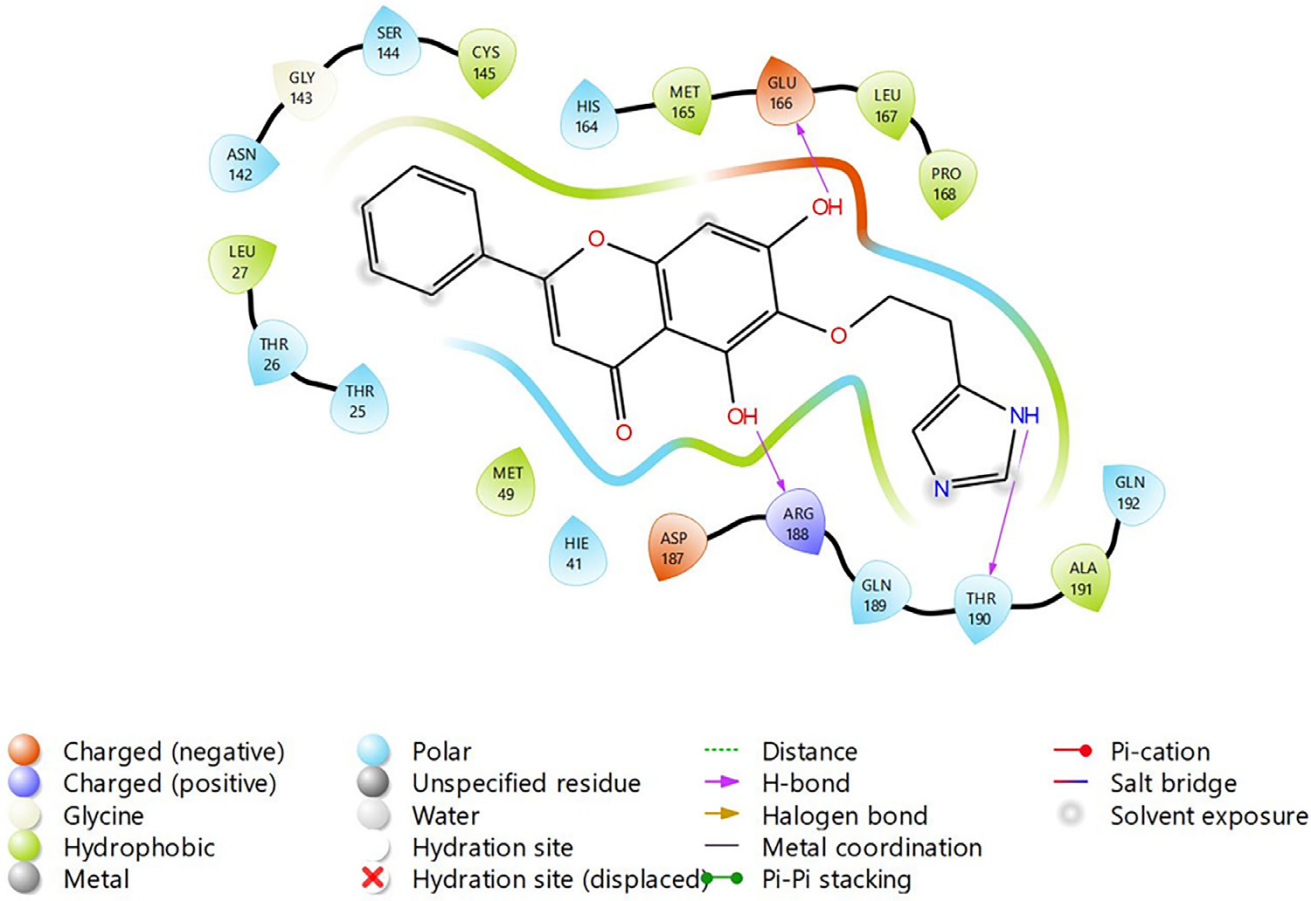
Binding interactions diagram of the top three stable virtual hit molecule 3 identified from the SP docking method at the SARS-CoV-2 M^pro^ targetʼs binding site region (PDB ID: 6M2N), the substituted baicalein analogues designed based on the chemical enumeration of site 2.

**Fig. 26:**
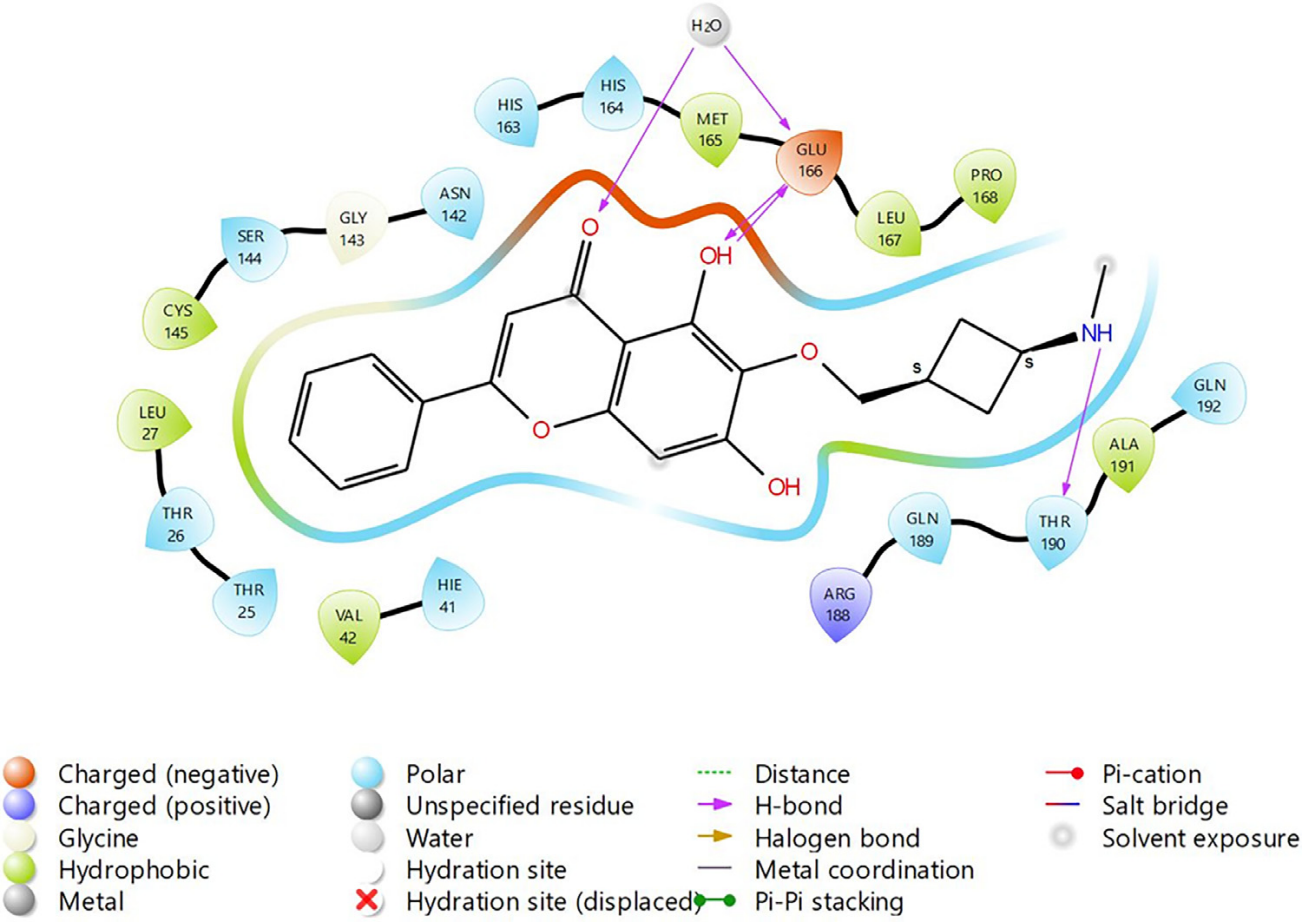
Binding interactions diagram of the top three stable virtual hit molecule 1 identified from the XP docking method at the SARS-CoV-2 M^pro^ targetʼs binding site region (PDB ID: 6M2N), the substituted baicalein analogues designed based on the chemical enumeration of site 2.

**Fig. 27:**
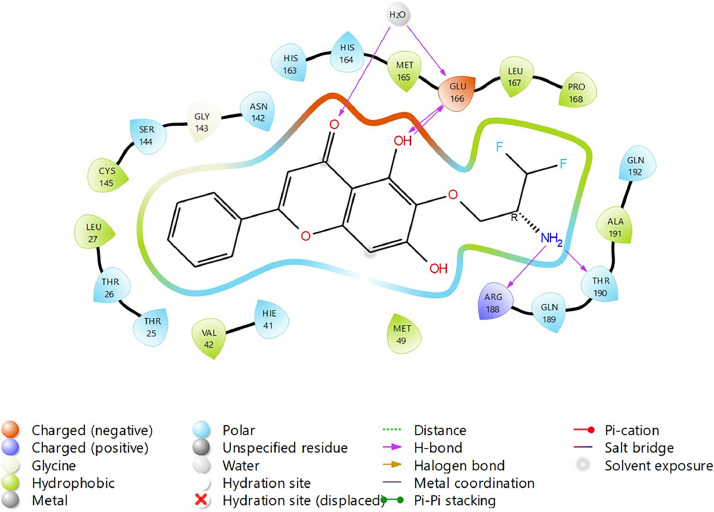
Binding interactions diagram of the top three stable virtual hit molecule 2 identified from the XP docking method at the SARS-CoV-2 M^pro^ targetʼs binding site region (PDB ID: 6M2N), the substituted baicalein analogues designed based on the chemical enumeration of site 2.

**Fig. 28:**
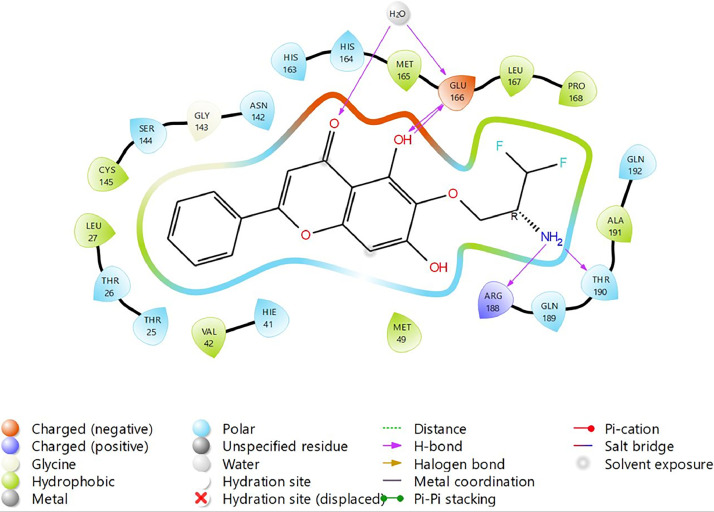
Binding interactions diagram of the top three stable virtual hit molecule 3 identified from the XP docking method at the SARS-CoV-2 M^pro^ targetʼs binding site region (PDB ID: 6M2N), the substituted baicalein analogues designed based on the chemical enumeration of site 2.

**Fig. 29:**
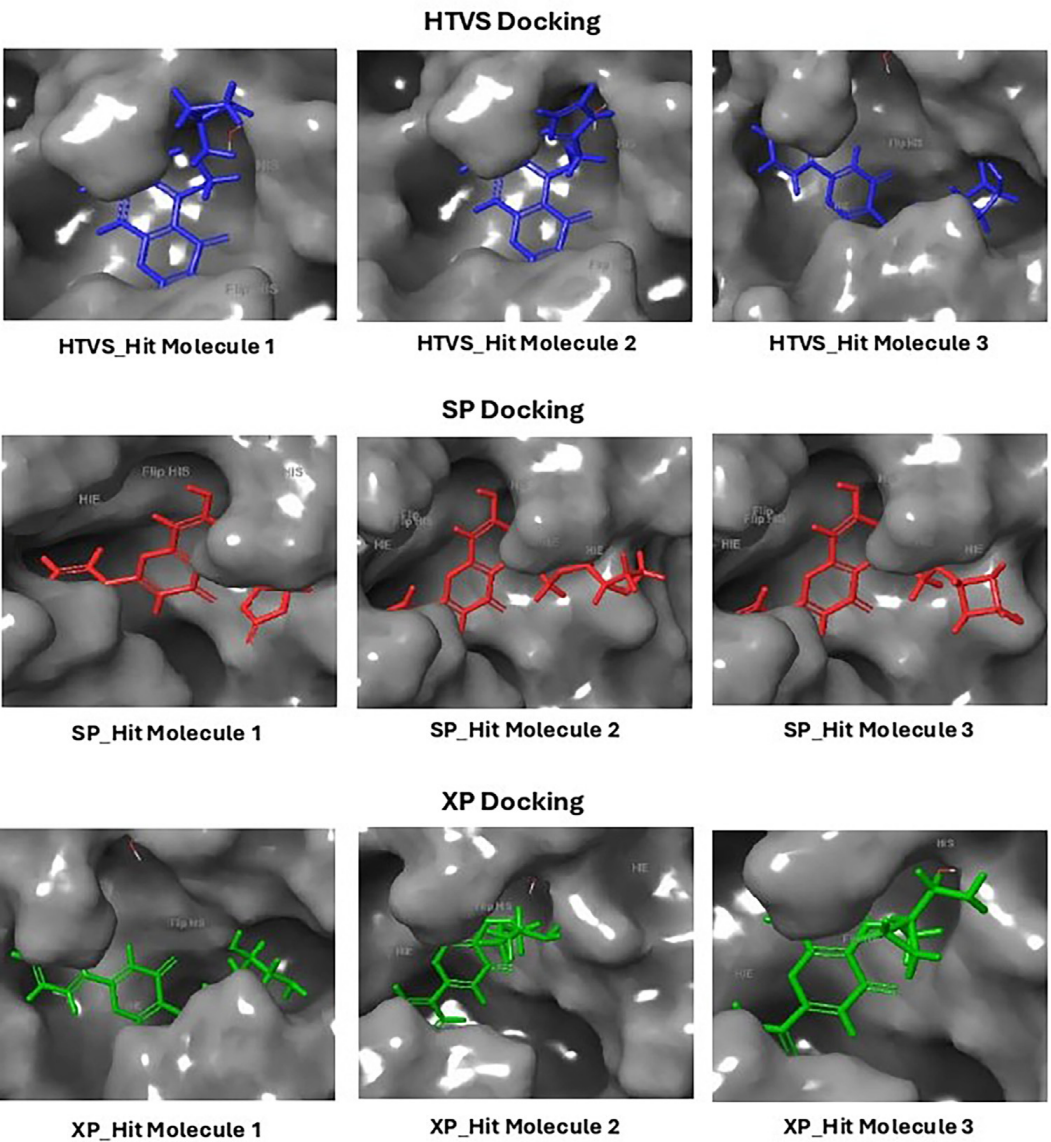
Binding orientation of the top three stable virtual hit molecules identified from HTVS, SP, and XP docking methods at the SARS-CoV-2 M^pro^ targetʼs binding site region (PDB ID: 6M2N), the substituted baicalein analogues designed based on the chemical enumeration of site 3.

**Fig. 30:**
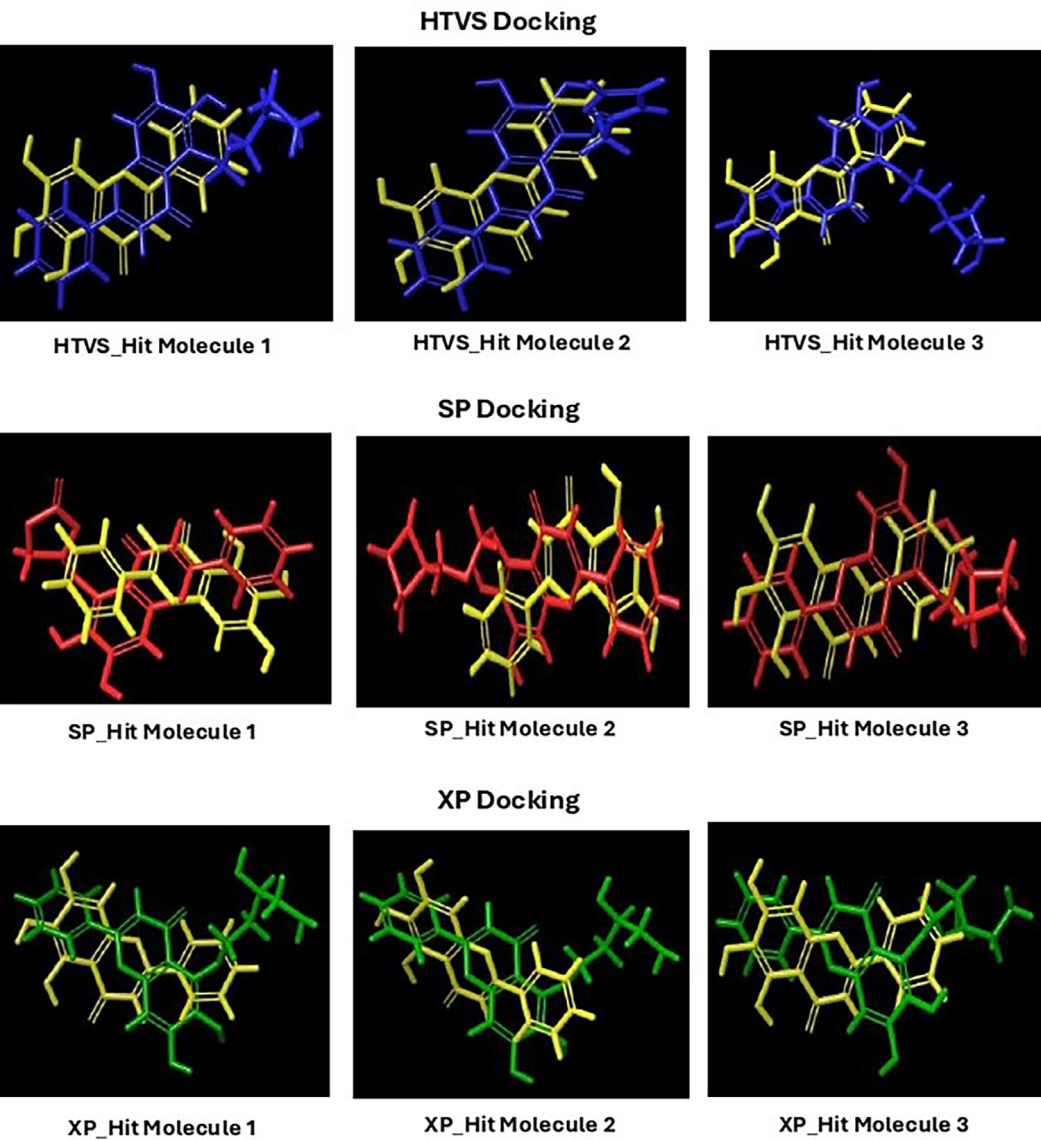
Superimposed stable binding conformations of the baicalein and the top three stable virtual hit molecules identified from HTVS, SP, and XP docking methods at the SARS-CoV-2 M^pro^ targetʼs binding site region (PDB ID: 6M2N), the substituted baicalein analogues designed based on the chemical enumeration of site 3.

**Fig. 31:**
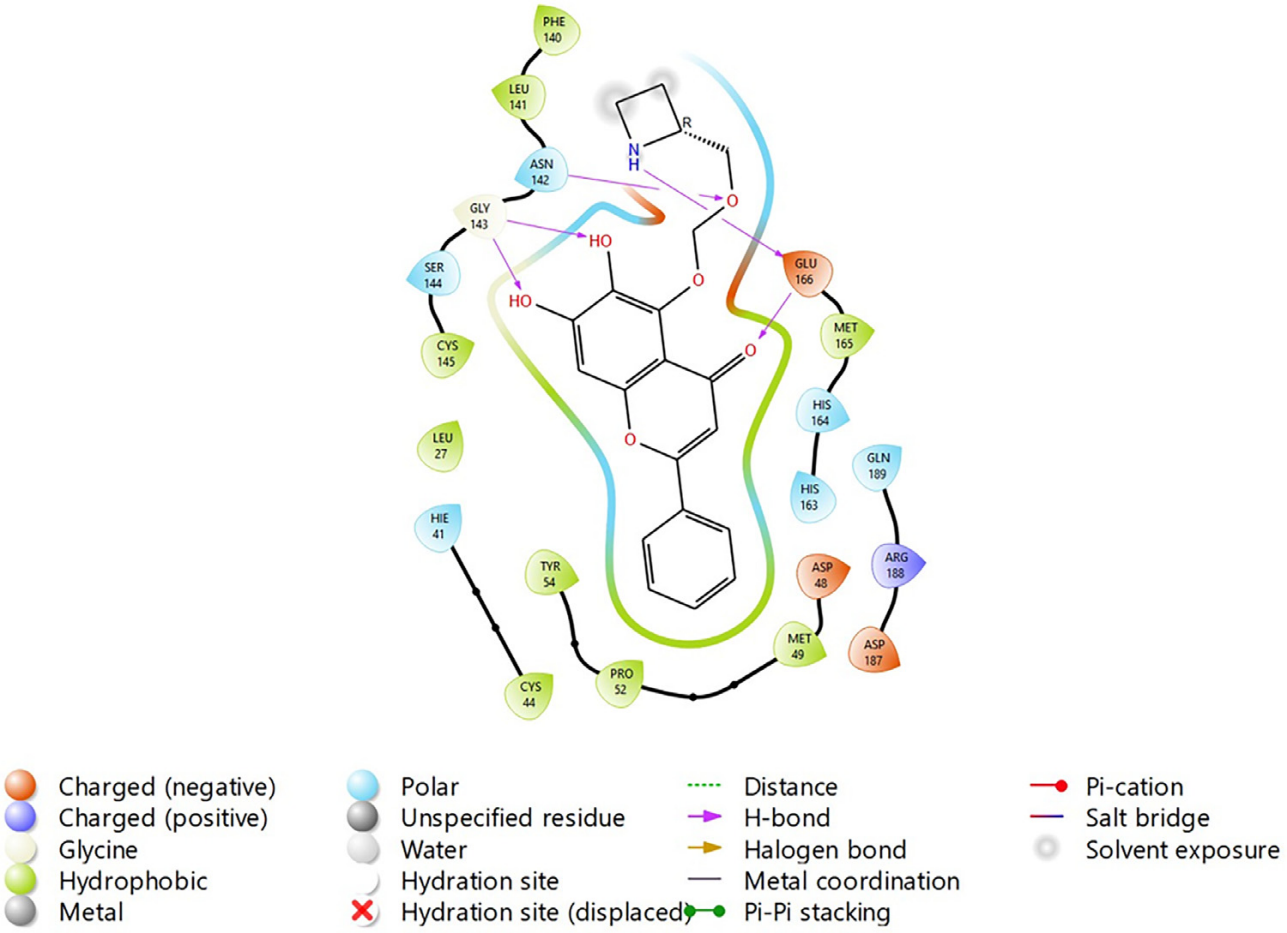
Binding interactions diagram of the top three stable virtual hit molecule 1 identified from the HTVS docking method at the SARS-CoV-2 M^pro^ targetʼs binding site region (PDB ID: 6M2N), the substituted baicalein analogues designed based on the chemical enumeration of site 3.

**Fig. 32:**
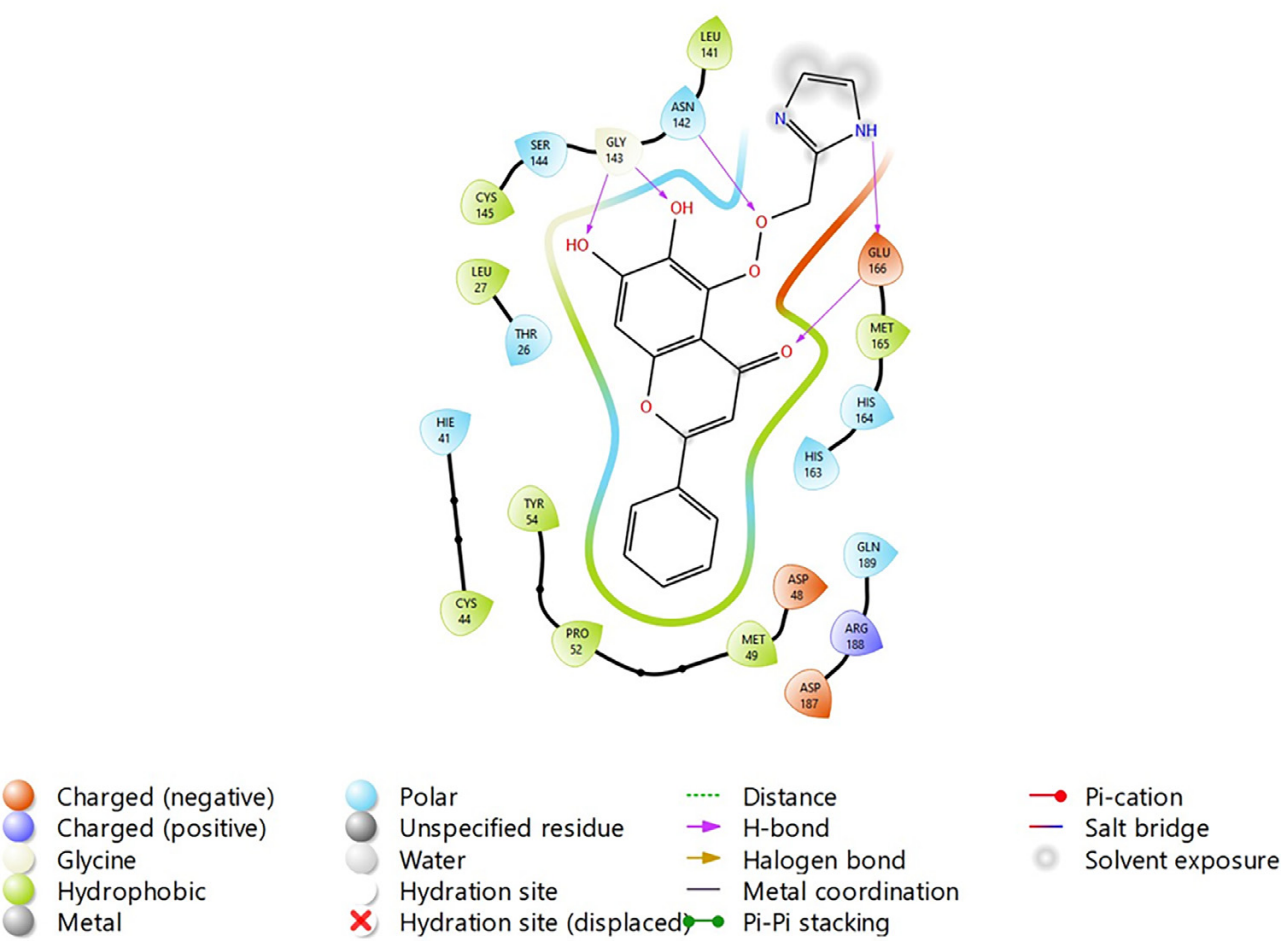
Binding interactions diagram of the top three stable virtual hit molecule 2 identified from the HTVS docking method at the SARS-CoV-2 M^pro^ targetʼs binding site region (PDB ID: 6M2N), the substituted baicalein analogues designed based on the chemical enumeration of site 3.

**Fig. 33:**
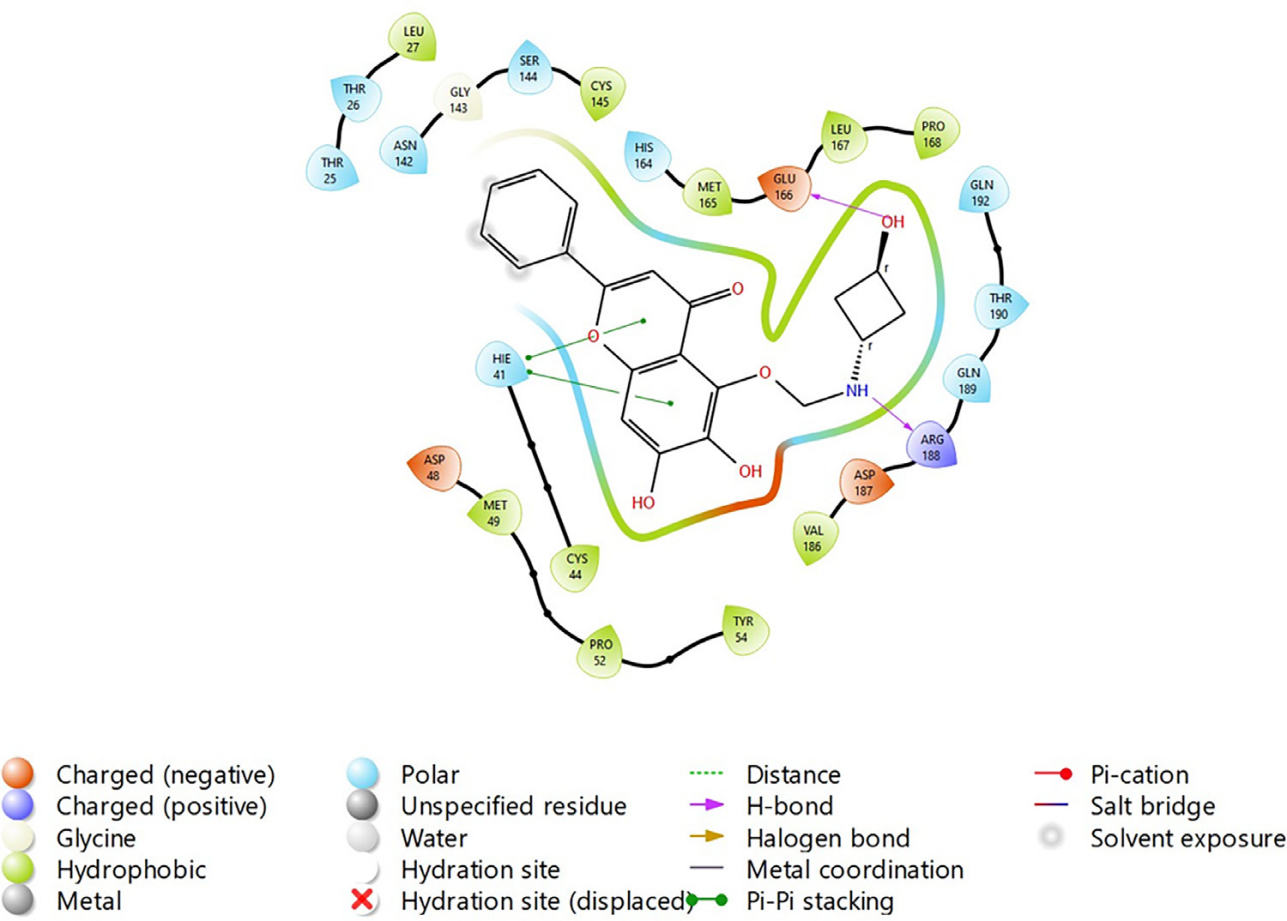
Binding interactions diagram of the top three stable virtual hit molecule 3 identified from the HTVS docking method at the SARS-CoV-2 M^pro^ targetʼs binding site region (PDB ID: 6M2N), the substituted baicalein analogues designed based on the chemical enumeration of site 3.

**Fig. 34:**
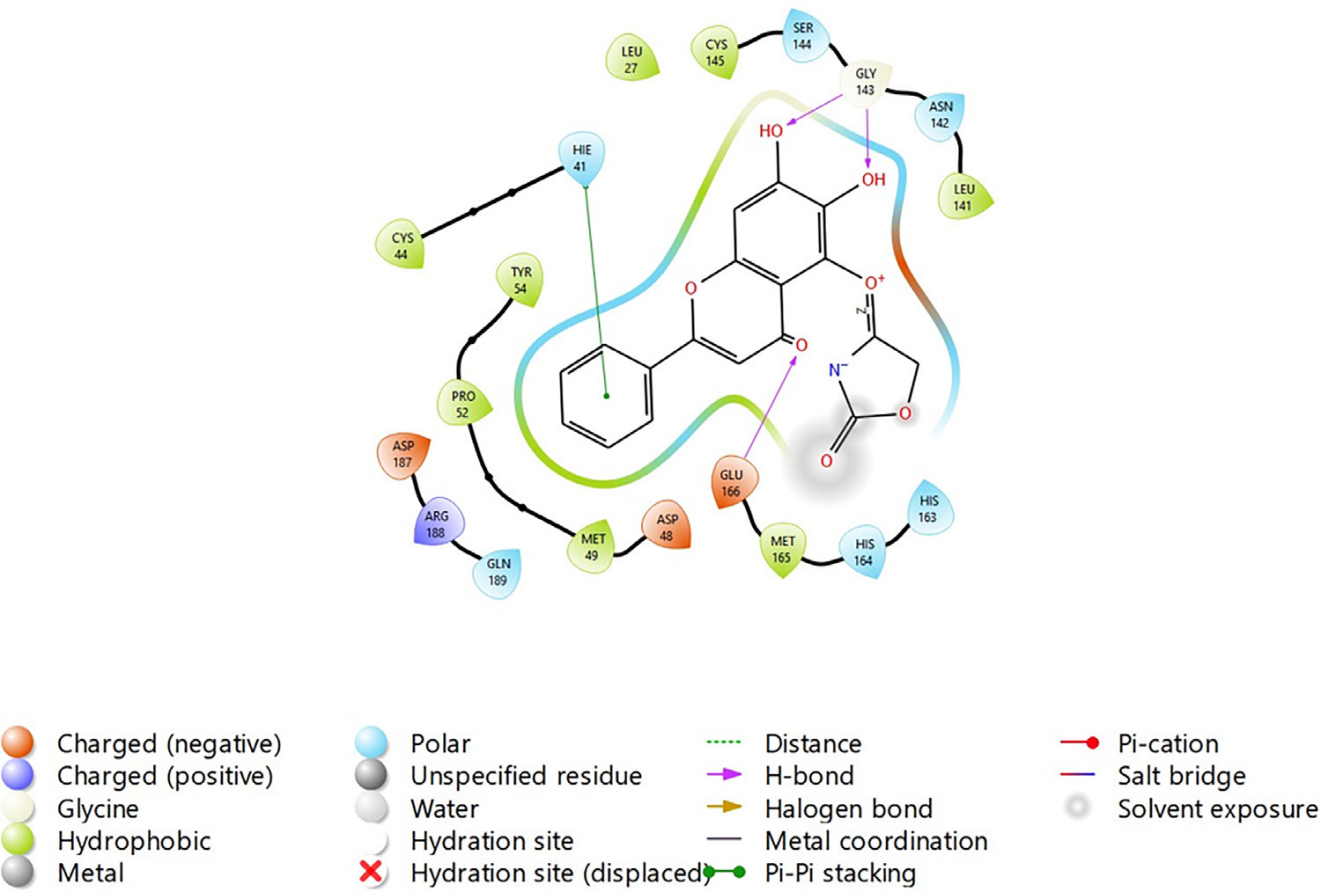
Binding interactions diagram of the top three stable virtual hit molecule 1 identified from the SP docking method at the SARS-CoV-2 M^pro^ targetʼs binding site region (PDB ID: 6M2N), the substituted baicalein analogues designed based on the chemical enumeration of site 3.

**Fig. 35:**
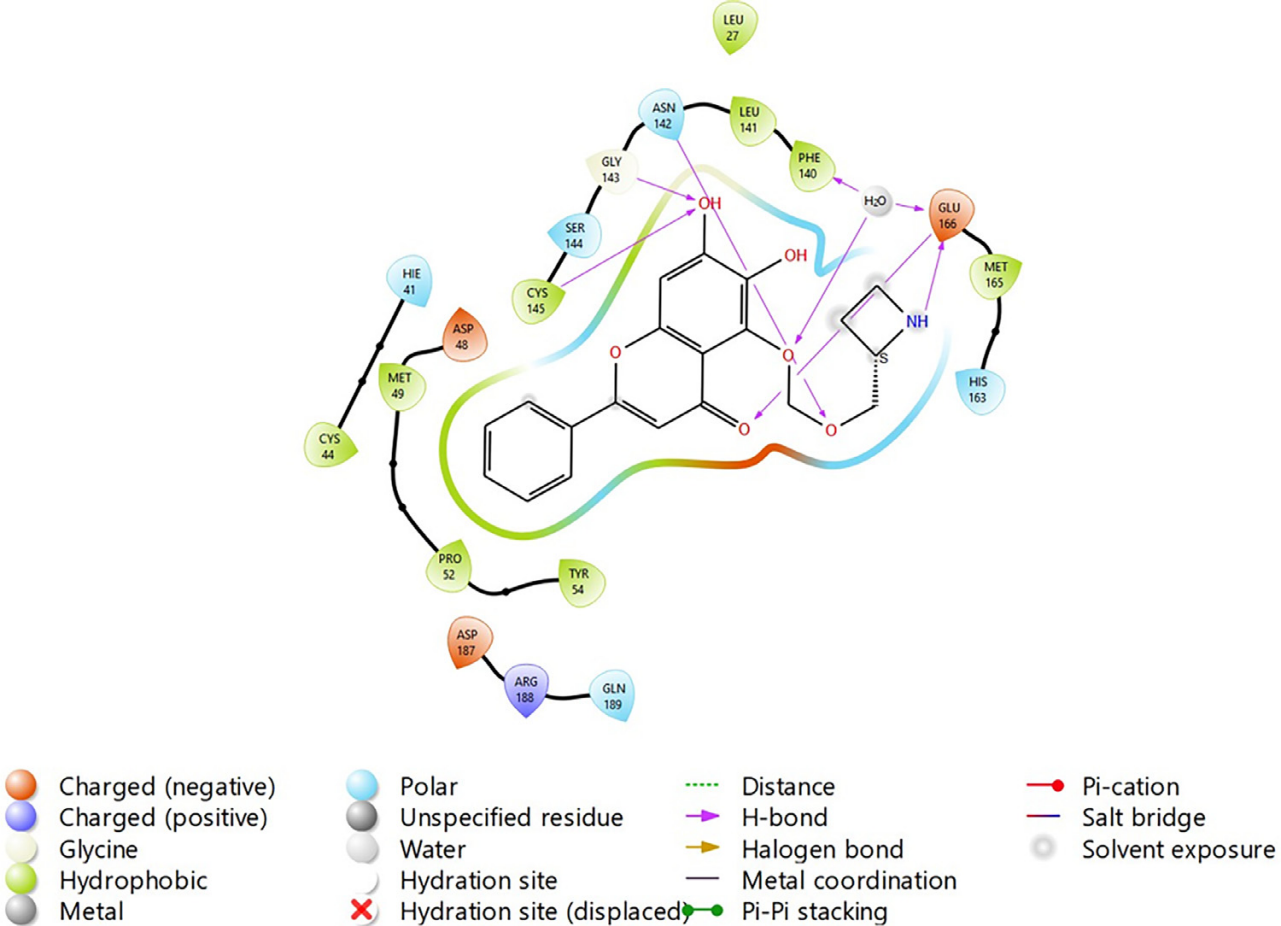
Binding interactions diagram of the top three stable virtual hit molecule 2 identified from the SP docking method at the SARS-CoV-2 M^pro^ targetʼs binding site region (PDB ID: 6M2N), the substituted baicalein analogues designed based on the chemical enumeration of site 3.

**Fig. 36:**
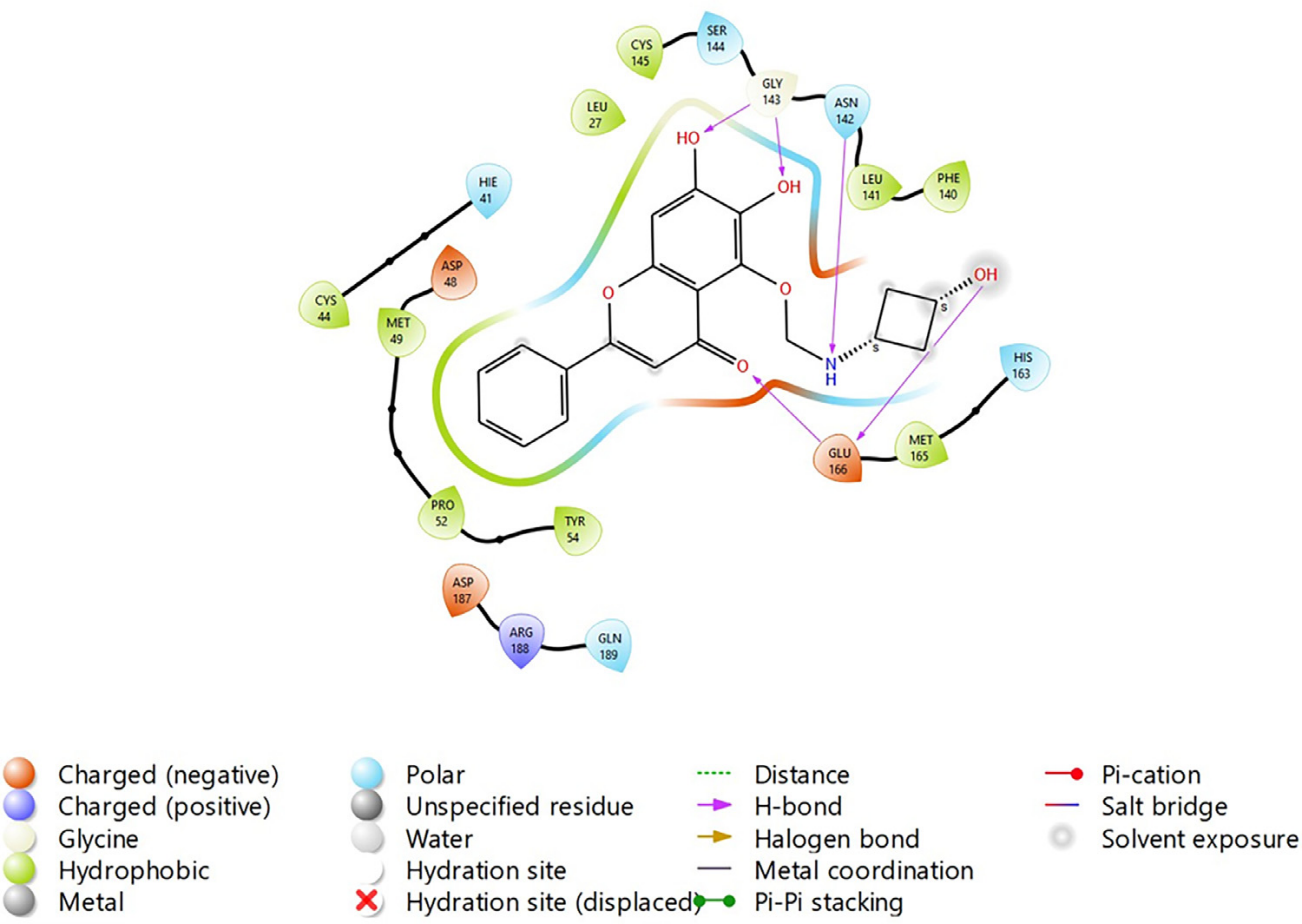
Binding interactions diagram of the top three stable virtual hit molecule 3 identified from the SP docking method at the SARS-CoV-2 M^pro^ targetʼs binding site region (PDB ID: 6M2N), the substituted baicalein analogues designed based on the chemical enumeration of site 3.

**Fig. 37:**
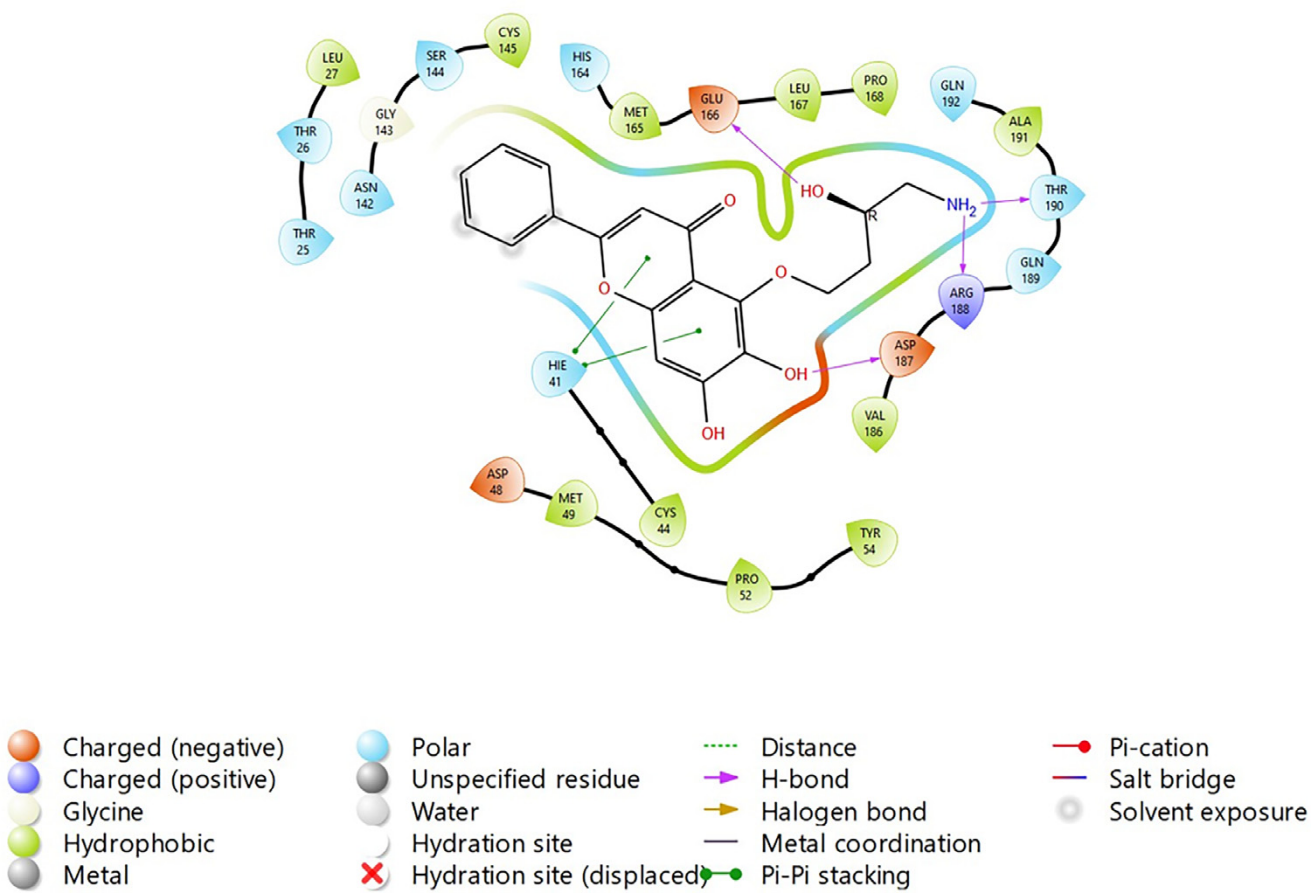
Binding interactions diagram of the top three stable virtual hit molecule 1 identified from the XP docking method at the SARS-CoV-2 M^pro^ targetʼs binding site region (PDB ID: 6M2N), the substituted baicalein analogues designed based on the chemical enumeration of site 3.

**Fig. 38:**
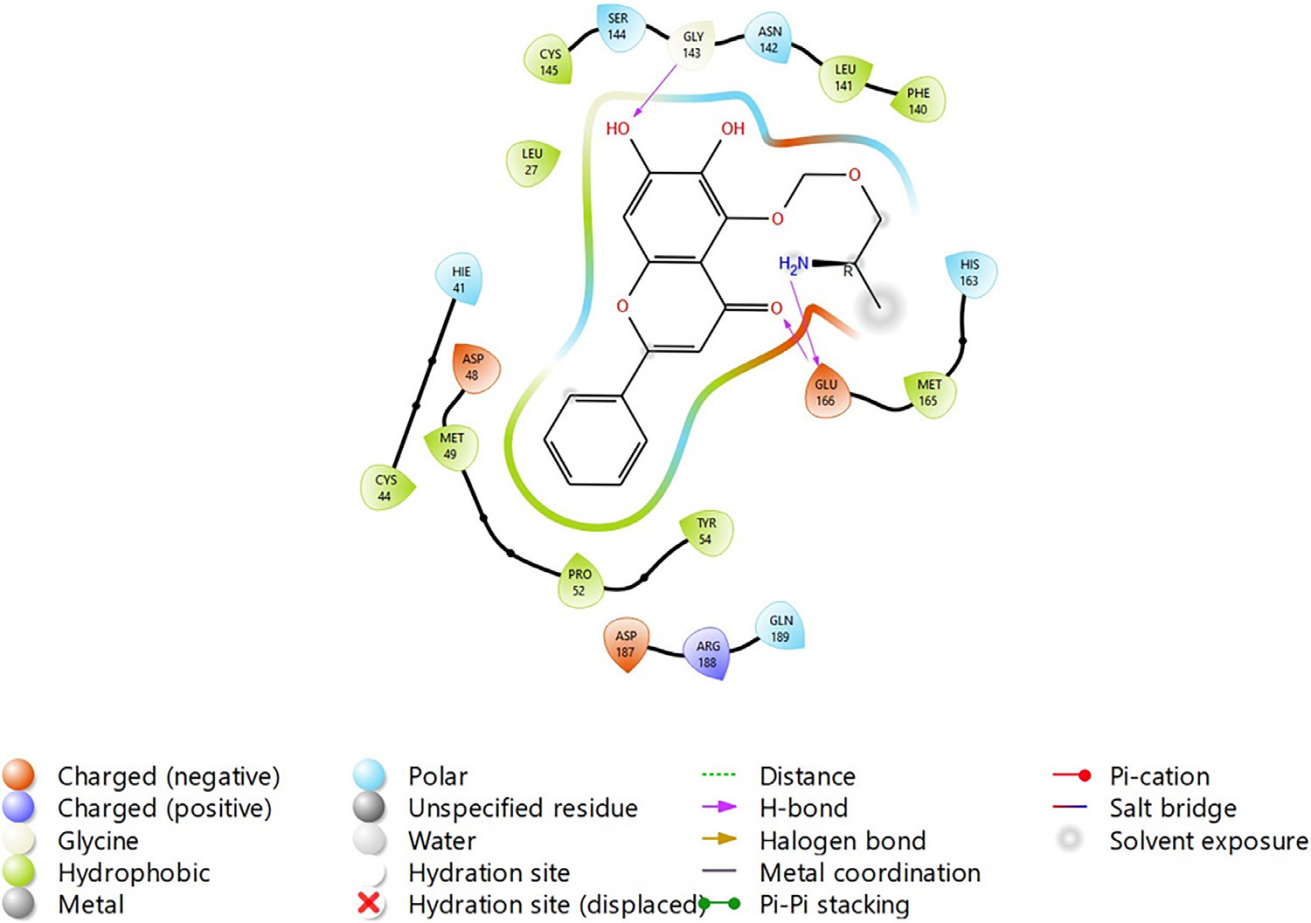
Binding interactions diagram of the top three stable virtual hit molecule 2 identified from the XP docking method at the SARS-CoV-2 M^pro^ targetʼs binding site region (PDB ID: 6M2N), the substituted baicalein analogues designed based on the chemical enumeration of site 3.

**Fig. 39:**
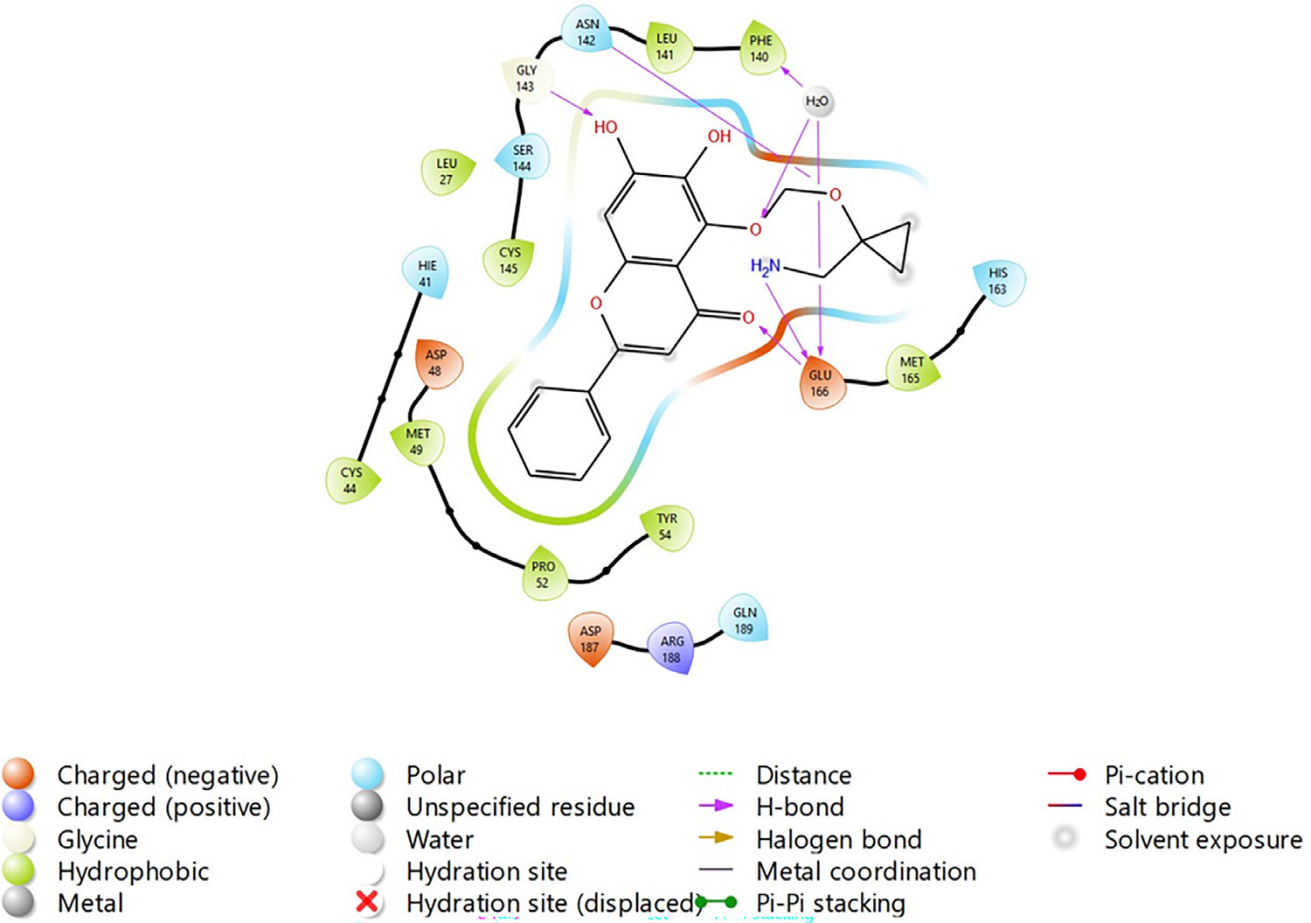
Binding interactions diagram of the top three stable virtual hit molecule 3 identified from the XP docking method at the SARS-CoV-2 M^pro^ targetʼs binding site region (PDB ID: 6M2N), the substituted baicalein analogues designed based on the chemical enumeration of site 3.

Based on the co-crystallized bioactive ligand conformation of baicalein, 2563 baicalein analogues are generated for each of the three different enumeration sites (Fig. 1). Virtual screening of the analogues against SARS-CoV-2 M^pro^ was done using HTVS, SP, and XP docking methods to reveal the docking scores of each analogue. The first three stable analogues with the lowest docking score for each enumeration site are tabulated in Tables 2 (site 1), Table 3 (site 2), and Table 4 (site 3), respectively. It can be easily observed that the docking scores of tabulated analogues are lower compared to the binding properties of standard baicalein shown in Table 1 and Fig. 3, Fig. 4, Fig. 5, Fig. 6. The important amino acid residues for the binding pocket of SARS-CoV-2 M^pro^ include His 41, Cys 44, Met 49, Leu 141, Asn 142, Gly 143, Ser 144, Cys 145, His 164, Met 165, Glu 166, Asp 187, and Gln 189. The 2D representation is shown in Fig. 2. Besides, the interaction between the potent analogues and essential amino acid residues of SARS-CoV-2 M^pro^ is shown in the 2D diagrams shown as Figures, based on the individual sites of enumeration, for the most stable analogues of site 1 (Fig. 9, Fig. 10, Fig. 11, Fig. 12, Fig. 13, Fig. 14, Fig. 15, Fig. 16, Fig. 17), site 2 (Fig. 20, Fig. 21, Fig. 22, Fig. 23, Fig. 24, Fig. 25, Fig. 26, Fig. 27, Fig. 28), and site 3 (Fig. 31, Fig. 32, Fig. 33, Fig. 34, Fig. 35, Fig. 36, Fig. 37, Fig. 38, Fig. 39), respectively. Based on the binding orientations and superimposed conformational analysis of Site 1 shown in Fig. 7, Fig. 8, Site 2 shown in Fig. 18, Fig. 19, and Site 3 shown in Fig. 29, Fig. 30, it is obvious that all identified baicalein analogues had similar binding modes. In addition, it also revealed the formation of hydrogen bonds with Glu 166 as well as other hydrogen bonds with some amino acid residues of M^pro^ binding site. For example, the rank 1 baicalein analogues under XP docking in Table 2 form 5 hydrogen bonds with the active site amino acids, and it showed the lowest docking score of −11.905 kcal/mol against SARS-CoV-2 M^pro^ among all the potent analogues, showing that it has the highest binding affinity. The result is inspiring as, compared to these analogues, standard baicalein exhibits only two hydrogen bond interactions and a much lower binding affinity, with a docking score of −8.005 kcal/mol even using an XP docking method.

**Table 1:** Binding properties of the baicalein docked using HTVS, SP, and XP methods at the SARS-CoV-2 M^pro^ targetʼs binding site region (PDB ID: 6M2N).

Chemical structure of the baicalein	Docking method	Binding energy (kcal/mol)	Number of hydrogen bonds	Hydrogen bond interacting residues
	HTVS	−7.271	3	Gly 143, Glu 166
	SP	−7.829	3	Gly 143, Glu 166
	XP	−8.005	3	Gly 143, Glu 166

**Table 2:** Binding properties of the top three stable virtual hit molecule identified from HTVS, SP, and XP docking methods at the SARS-CoV-2 M^pro^ target's binding site region (PDB ID: 6M2N), the substituted baicalein analogues designed based on the chemical enumeration of site 1.

Docking method	Chemical structure of the stable virtual hit molecule	Binding energy (kcal/mol)	Number of hydrogen bonds	Hydrogen bond interacting residues	RMSD from baicalein orientation
HTVS		−9.722	4	Gly 143, Glu 166, Cys 145, Thr 26	4.8039

		−9.497	5	Gly 143, Glu 166, Cys 145, Thr 26	4.6377

	−9.420	4	Gly 143, Glu 166, Asn 142	4.6480

SP	*	−10.266	5	Gly 143, Glu 166, Thr 26, Asn 142	4.5722
		−9.906	4	Gly 143, Glu 166, Thr 26, Asn 142	4.6403

	−9.896	4	Gly 143, Glu 166, Thr 26, Asn 142	4.5690

XP		−11.905	5	Gly 143, Glu 166, Asn 142, Thr 26	4.4470

	*	−11.790	5	Gly 143, Glu 166, Asn 142	4.7048

	−11.714	4	Gly 143, Glu 166, Thr 26	4.4658

⁎ A virtual hit molecule is identified as a stable ligand in more than one docking method; RMSD: Root Mean Square Deviation.

**Table 3:** Binding properties of the top three stable virtual hit molecule identified from HTVS, SP, and XP docking methods at the SARS-CoV-2 M^pro^ targetʼs binding site region (PDB ID: 6M2N), the substituted baicalein analogues designed based on the chemical enumeration of site 2.

Docking method	Chemical structure of the stable virtual hit molecule	Binding energy (kcal/mol)	Number of hydrogen bonds	Hydrogen bond interacting residues	RMSD from baicalein orientation
HTVS		−8.212	4	Glu 166, Arg 188, Thr 190	4.6146

		−7.985	2	Arg 188, Thr 190	4.6045

	−7.828	4	Glu 166, Arg 188, Thr 190	4.6300

SP		−8.750	3	Glu 166, Arg 188, Thr 190	4.5301
	*	−8.679	4	Glu 166, Arg 188, Thr 190	4.5204

	−8.556	3	Glu 166, Arg 188, Thr 190	4.5307

XP		−9.980	3	Glu 166, Thr 190	4.7853

	*	−9.963	4	Glu 166, Arg 188, Thr 190	4.7813

	−9.944	4	Glu 166, Arg 188, Thr 190	4.7924

⁎ A virtual hit molecule is identified as a stable ligand in more than one docking method; RMSD: Root Mean Square Deviation.

**Table 4:** Binding properties of the top three stable virtual hit molecule identified from HTVS, SP, and XP docking methods at the SARS-CoV-2 M^pro^ targetʼs binding site region (PDB ID: 6M2N), the substituted baicalein analogues designed based on the chemical enumeration of site 2.

Docking method	Chemical structure of the stable virtual hit molecule	Binding energy (kcal/mol)	Number of hydrogen bonds	Hydrogen bond interacting residues	RMSD from baicalein orientation
HTVS	*	−8.821	5	Gly143, Glu 166, Asn 142	4.7156

		−8.698	5	Gly143, Glu 166, Asn 142	4.7073

*	−8.606	2	Glu 166, Arg 188	4.7214
SP		−9.263	3	Gly 143, Glu 166	4.6129

	*	−9.211	5	Gly 143, Glu 166, Asn 142, Cys 146	4.7062

*	−9.092	5	Gly 143, Glu 166, Asn 142	4.6154
XP		−10.557	4	Glu 166, Arg 188, Asp 187, Thr 190	4.7248

		−10.329	3	Gly 143, Glu 166	4.6701

	−10.229	4	Gly143, Glu 166, Asn 142	4.7248


⁎ A virtual hit molecule is identified as a stable ligand in more than one docking method; RMSD: Root Mean Square Deviation.

The docking studies [10] revealed that the rutin formed hydrogen boding interactions with Leu141 (1.91 Å), Cys145 (2.54 Å), and Glu166 (2.57 Å) respectively at the catalytic binding domains of M^pro^. There was another study conducted by Himanshu Rai et al. on a set of drugs that include chloroquine (CQ), hydroxychloroquine (HCQ), remdesivir (RDV), arbidol (ARB), and glycyrrhizin (GA) [11]. CQ formed hydrogen bonds with Gln192 (2.16 Å) and Thr190 (2.70 Å), Glu166 (1.73 Å) and His164 (3.60 Å). Besides, alkyl hydrophobic interactions with Pro168 (3.87 Å) and Cys145 (5.46 Å and 4.81 Å). CQ also formed mixed π-alkyl hydrophobic interactions with M165 (4.52 Å), Pro168 (5.15 Å), and His163 (4.29 Å). These interactions allow the high binding affinity of CQ to M^pro^ by forming strong ligand stability at the target binding site with a docking score of −6.41 kcal/mol. In terms of HCQ, hydrogen bond interactions were formed with Ser144 (2.94 Å), Leu141 (2.05 Å), and Cys145 (2.31 Å). It also exhibited π-sigma hydrophobic interaction, π-π interaction, and π -alkyl hydrophobic interaction with Met165 (3.90/4.22 Å), His41 (4.84 Å), and His163 (5.27 Å) respectively. These interactions allow the high binding affinity of HCQ to M^pro^ by forming strong ligand stability at the target binding site with a docking score of −6.01 kcal/mol. RDV showed hydrogen bonds with Gln192 (1.81 Å), Thr190 (2.06 Å and 1.87 Å), and Glu166 (1.73 Å). There were also carbon-hydrogen bonds formed with Glu166 (3.01 Å). His41 (4.83 Å) and Pro168 (4.63 Å) formed π-alkyl hydrophobic interaction with RDV. The binding score of RDV was −7.12 kcal/ mole, making it a potent inhibitor against M^pro^. ARB formed the hydrogen bond with His164 (2.11 Å), His164 (1.81 Å), and Cys145 (2.60 Å). Glu166 contributed two π-donor hydrogen bonds of 2.55 Å and 3.77 Å. His 164 formed a weak conventional hydrogen bond and a π-sigma hydrophobic interaction with bond lengths of 4.26 Å and 4.25 Å respectively. Alkyl hydrophobic interactions were observed at His163 (5.04 Å) and Cys145 (4.68), together with π-alkyl hydrophobic interaction at Leu167 (4.63 Å) and π-sigma hydrophobic interaction with Pro168 (4.63 Å). Cys145 (5.75 Å) also formed one π-sulfur interaction with M^pro^. The docking score for ARB is − 7.0 kcal/ mol, indicating a high affinity towards the catalytic binding site of M^pro^. GA formed hydrogen bonds with Glu166 (2.06 Å), Phe140 (1.91 Å), Ser144 (2.76 Å), and Gln189 (3.01 Å and 2.19 Å). Besides, Cys145 (4.48 Å), Met49 (4.94 and 4.99 Å), Met165 (4.62 and 5.03 Å), and Pro168 (4.88 Å) exhibited hydrophobic alkyl interactions. The docking score of GA is −8.21 kcal/mol, which is the highest negative value among the set of investigated drugs.

The computational study by Anas Shamsi et al. [12] identified glecaprevir and maraviroc (MVC) as the most effective SARS-CoV-2 M^pro^ inhibitors. The formation of three hydrogen bond interactions between Thr25, Cys145, and Gln189, together with two fluorine interactions with Thr26 and Gly143 at the binding pocket of M^pro^ is among the significant interactions exhibited by glecaprevir. MVC engaged with specific amino acids of M^pro^ similarly, forming four hydrogen connections with Ser46, Phe140, Cys145, and Glu166. Besides, there was a fluorine interaction with Thr26. glecaprevir and MCV exhibited similar binding interactions, and both of them were found to be well-suited to the deep cavity of the M^pro^ catalytic binding site to obstruct substrate accessibility and hence decrease its enzymatic activity.

The docking study by Akshita Gupta et al. [13] suggested that 6-(adenosine tetraphosphate-methyl)-7,8-dihydropterin (DB04158), NADPH (DB02238), denufosol (DB04983), and 4-oxo-nicotinamide-adenine dinucleotide phosphate (DB01753) are the highly potential drugs against M^pro^ as they gave high glide energy and docking score. The drug DB04158 formed a complex with M^pro^ with a docking score of −9.958 kcal/mol and glide energy of −110.38 kcal/mol. Hydrogen bond formations were observed in Glu47, Asn142, His164, Glu166, and Thr190. Hydrophobic interactions were exhibited in Thr24, Thr25, Thr26, His41, Cys145, Pro168, Arg188, and Gln189. The amino acid residues His41 and Cys145 in the catalytic dyad engage with NADPH via hydrogen bonds and hydrophobic interactions respectively. The docking score of this complex is −10.303 kcal/mol with a glide energy of −100.345 kcal/mol. Besides His41, other amino acid residues including Thr25, Cys44, Asn142, Glu166, Arg188, and Gln189 were also involved in the formation of hydrogen bonds. Hydrophobic interactions with NADPH were also facilitated by other amino acids residues Leu27, Val42, Thr45, Ser46, Met49, Leu141, Cys145, His163, His164, Pro168, and Gln192. Denufosol has a docking score of −11.154 kcal/mol and a glide energy of −89.690 kcal/mol. The hydrogen bond formations in the complex were observed in Thr25, Thr26, Glu47, Asn142, Glu166, Gln189, and Thr190. Strong hydrophobic interactions that provided stability of the complex include amino acid residues His41, Met49, Ser139, Leu141, Asn142, Ser144, Cys145, His163, Leu167, Asp187, Arg188, and Gln189. The drug DB01753 formed a complex with M^pro^ with a docking score of −11.154 kcal/mol and glide energy of −89.690 kcal/mol. Hydrogen bonds formation were observed in Cys44, Phe140, Asn142, Glu166, and Gln189, whereas hydrophobic interactions were seen in His41, Glu47, Met49, Phe140, Leu141, Ser144, Cys145, His163, Pro168, His172, and Ala191.

In terms of the results for the investigation on baicalein analogues in this study, we identified Glu166, Gly143, Arg188, Asn142, Thr26, and Thr190 are the crucial amino acid residues in forming hydrogen bond interactions at the catalytic binding site of M^pro^ and ligands with reported antiviral properties. Similar observations were revealed based on the study conducted by Himanshu Rai et.al, amino acids such as Cys145, Glu166, and His163 predicted as specific to M^pro^ that interact with the target molecules. Likewise, the investigation by Anas Shamsi et al. on Glecaprevir and MVC suggested that Cys145 and Thr26 are the critical amino acid residues to form hydrogen bonding interactions within the target site of M^pro^. In addition, the study done by Akshita Gupta et al. suggested that there were a variety of amino acids residues forming interactions with a set of drugs include Glu47, Asn142, His164, Glu166, and Thr190, Thr25, Cys44, Arg188, and Gln189, His41, Thr26, and Phe140, and among these residues, Asn142 and Glu166 were indicated as the most essential ones as they formed hydrogen bonds with each one of the drugs respectively.

## 5 Summary

The lack of specific treatment for SARS-CoV-2 has led to extensive research in the discovery of prospective antiviral agents to tackle COVID-19. The potentiality of the baicalein analogues towards M^pro^ as drug candidates were proved by the molecular-based virtual screening study. The analogues of site 1 substitution ranked 1 in SP and ranked 2 in XP (S1, SP1, XP2), (S2, SP2, XP2), (S3, HTVS1, SP2) and (S3, HTVS3, SP2) showed repeated hits at the catalytic binding site of M^pro^. They exhibited lower docking scores as compared to standard baicalein in HTVS, SP, and XP docking methods. On top of that, the crucial amino acids in the formation of hydrogen bonds interaction were identified to be Glu166, Gly143, Arg188, Asn142, Thr26, and Thr190. This study has highlighted a subset of prospective baicalein analogues for further development as potential SARS-CoV-2 specific M^pro^ inhibitors.

## Limitations

None.

## Ethics Statement

The authors have read and follow the ethical requirements for publication in Data in Brief and confirming that the current work does not involve human subjects, animal experiments, or any data collected from social media platforms.

## CRediT authorship contribution statement

**Qiao Jie Wong:** Validation, Data curation, Writing – original draft, Visualization, Investigation, Software, Validation, Writing – review & editing. **Zhe Hong Low:** Validation, Data curation, Writing – original draft, Visualization, Investigation, Writing – review & editing. **Zi Yue Chan:** Validation, Data curation, Writing – original draft, Visualization, Investigation, Writing – review & editing. **Vasudeva Rao Avupati:** Conceptualization, Methodology, Software, Visualization, Investigation, Supervision, Writing – review & editing.

## Data Availability

Ligand-Target Interaction Diagrammes: Baicalein Analogues as SARS-CoV-2 Main Protease (Mpro) Inhibitors (Original data) (Mendeley Data)
